# Comparative effectiveness and safety of fluticasone-based versus beclometasone-based single-inhaler triple therapies in patients with chronic obstructive pulmonary disease: a population-based cohort study

**DOI:** 10.1007/s11096-025-02037-4

**Published:** 2025-11-07

**Authors:** Yaa-Hui Dong, Sheng-Wei Pan, Ming-Ching Chen, Chun-Yu Chen, Ning-Hsin Tsai, Hiraku Kumamaru

**Affiliations:** 1 Department of Pharmacy, College of Pharmaceutical Sciences, National Yang Ming Chiao Tung University, 155, Sec 2, Linong Street, Taipei, 112 Taiwan; 2 Institute of Public Health, School of Medicine, National Yang Ming Chiao Tung University, Taipei, Taiwan; 3 Department of Chest Medicine, Taipei Veterans General Hospital, Taipei, Taiwan; 4 Faculty of Medicine, School of Medicine, National Yang Ming Chiao Tung University, Taipei, Taiwan; 5 Department of Health Data Science, Graduate School of Data Science, Yokohama City University, Yokohama, Kanagawa Prefecture Japan; 6 Department of Healthcare Quality Assessment, Graduate School of Medicine, The University of Tokyo, Tokyo, Japan

**Keywords:** Adrenergic beta-2 receptor agonists, Cohort study, Glucocorticoids, Muscarinic antagonists, Pulmonary disease, chronic obstructive, Single-inhaler triple therapies

## Abstract

**Introduction:**

There is a paucity of comparative real-world evidence for fluticasone-based and beclometasone-based single-inhaler triple therapies in patients with chronic obstructive pulmonary disease (COPD).

**Aim:**

To compare clinical outcomes of fluticasone/umeclidinium/vilanterol (a once-daily dry powder inhaler) and beclometasone/glycopyrrolate/formoterol (a twice-daily metered dose inhaler) in patients with COPD.

**Method:**

This population-based cohort study enrolled patients with COPD who initiated fluticasone/umeclidinium/vilanterol or beclometasone/glycopyrrolate/formoterol from a nationwide Taiwanese database between 2019 and 2022. The effectiveness outcomes included severe and moderate exacerbations and the safety outcomes were pneumonia and composite cardiovascular events. Patients were followed from the first day after cohort entry to the earliest of each outcome occurrence, study treatment discontinuation or change, death, end of data (2022/12/31), or the 365th day after cohort entry. Cox regression models were employed to estimate hazard ratios (HRs) and corresponding 95% confidence intervals (CIs) for each outcome comparing fluticasone/umeclidinium/vilanterol versus beclometasone/glycopyrrolate/formoterol after high-dimensional propensity score matching.

**Results:**

There were 12,971 initiators included in the high-dimensional propensity score matched cohort. The HR suggested a lower risk of severe and moderate exacerbations (0.80 [95% CI 0.69–0.93] and 0.80 [95% CI 0.74–0.87], respectively) and a marginally non-significant decreased risk of pneumonia (0.85 [95% CI 0.70–1.02]) associated with fluticasone/umeclidinium/vilanterol. However, both treatments showed a similar risk of composite cardiovascular events (0.96 [95% CI 0.69–1.35]). The results were generally consistent across several pre-specified sensitivity and subgroup analyses. Of note, among patients treated for ≥ 90 days (nearly 73% of the initiators), the differences in clinical outcomes of both treatments tended to be minimal, with an HR of 0.98 (95% CI 0.78–1.23) for severe exacerbations, 0.93 (95% CI 0.72–1.20) for pneumonia, and 1.07 (95% CI 0.64–1.77) for composite cardiovascular events. Nevertheless, fluticasone/umeclidinium/vilanterol remained having a lower risk of moderate exacerbations (0.86 [95% CI 0.74–0.98]).

**Conclusion:**

This cohort study conducted in an Asian COPD population suggests that fluticasone/umeclidinium/vilanterol may be a preferred initial treatment option over beclometasone/glycopyrrolate/formoterol. While among patients who are able to maintain their therapies for ≥ 90 days, both treatments may demonstrate more comparable effectiveness and safety profiles.

**Supplementary Information:**

The online version contains supplementary material available at 10.1007/s11096-025-02037-4.

## Impact statements

- This population-based cohort study which comprehensively examined respiratory and cardiovascular outcomes of two single-inhaler triple therapies, fluticasone/umeclidinium/vilanterol (delivered via a dry powder inhaler) and beclometasone/glycopyrrolate/formoterol (delivered via a metered dose inhaler), suggests that fluticasone/umeclidinium/vilanterol may be a preferred initial treatment option in patients with COPD.
- The findings provide real-world evidence to inform treatment selection and also align with ongoing efforts to reduce greenhouse gas emissions from metered dose inhalers.
- On the other hand, by demonstrating more comparable benefits and risks of the two treatments among patients treated for ≥ 90 days, our results offer flexibility for individualized treatments, particularly for patients who experience difficulties with dry powder inhalers or who prefer metered dose inhalers.

## Introduction

Inhaled triple therapy, including inhaled corticosteroids (ICS), long-acting muscarinic antagonists (LAMA), and long-acting β2 agonists (LABA), has a unique role in chronic obstructive pulmonary disease (COPD) management [1]. Randomized controlled trials have found that triple therapies are more effective in lowering exacerbations than dual therapies in patients with an exacerbation history and a high symptom burden [2–4]. Currently, three single inhalers consisting of these triple therapies are marketed, including fluticasone-based (fluticasone/umeclidinium/vilanterol, a once-daily dry powder inhaler), budesonide-based (budesonide/glycopyrrolate/formoterol, a twice-daily metered dose inhaler), and beclometasone-based (beclometasone/glycopyrrolate/formoterol, a twice-daily metered dose inhaler) treatments [5–7].

Given the different physicochemical and pharmacokinetic profiles of individual ICS, LAMA, and LABA, clinical outcomes may vary across these triple therapies [8–11]. One network meta-analysis of clinical trials suggested that fluticasone/umeclidinium/vilanterol may have a lower risk of moderate-to-severe exacerbations than budesonide/glycopyrrolate/formoterol or beclometasone/glycopyrrolate/formoterol. However, this study included patients with various characteristics across trials and the indirect comparison may be prone to bias [12]. There are no head-to-head clinical trials of single-inhaler triple therapies, and the comparative observational studies remain limited. One U.S. cohort study including patients under the Medicare Fee-For-Service program found that fluticasone/umeclidinium/vilanterol was associated with a decreased risk of moderate-to-severe exacerbations than budesonide/glycopyrrolate/formoterol (hazard ratio [HR], 0.90; 95% confidence interval [CI] 0.87–0.93) [13]. Another U.S. cohort study analyzing commercial healthcare data also observed a reduced risk of moderate-to-severe exacerbations (HR 0.92 [0.88–0.96]) and severe exacerbations (HR 0.78 [0.68–0.89]) but a similar risk of pneumonia (HR 1.00 [0.91–1.10]) comparing fluticasone/umeclidinium/vilanterol to budesonide/glycopyrrolate/formoterol [14]. Notably, long-acting bronchodilators may have concerns about an increased cardiovascular risk [15, 16]. However, the two observational studies did not examine the cardiovascular safety profiles of both single-inhaler therapies. Moreover, although fluticasone/umeclidinium/vilanterol and budesonide/glycopyrrolate/formoterol are available in most countries, beclometasone/glycopyrrolate/formoterol is only marketed in several European and Asian countries but not in the U.S. There is still a paucity of real-world evidence comparing fluticasone/umeclidinium/vilanterol and beclometasone/glycopyrrolate/formoterol.

### Aim

Fluticasone/umeclidinium/vilanterol and beclometasone/glycopyrrolate/formoterol were both reimbursed in Taiwan in 2019–2022. Using real-world healthcare data, the present cohort study aimed to compare clinical outcomes of the two treatments in patients with COPD.

## Method

### Data source

The data source for this study was the Taiwan National Health Insurance Database (NHIRD), which consists of de-identified demographic and healthcare data—including diagnosis, procedure, and pharmacy dispensing claims records—from outpatient visits, emergency department visits, and hospital admissions covering approximately 23 million Taiwanese recruited in a single-payer, compulsory national health insurance system [17, 18].

### Study cohort who initiated single-inhaler triple therapies

The study population included patients with COPD who initiated fluticasone/umeclidinium/vilanterol or beclometasone/glycopyrrolate/formoterol from the Taiwan NHIRD between 2019/01/01 and 2022/12/31. Patients with COPD were identified using International Classification of Diseases, Tenth Revision diagnoses codes of J41-J44 in any diagnosis positions in the claims of outpatient visits, emergency department visits, or hospital admissions [19]. Receiving either single-inhaler triple therapy was ascertained using outpatient pharmacy dispensing claims (see Supplementary Table 1 for codes). The index date was the date of the first dispensing of a single-inhaler triple therapy following a diagnosis of COPD. Patients should have no dispensing of any study medications within 365 days preceding the index date. We further applied several exclusion criteria stated in Supplementary Method.

### Outcome measurement within one year

Previous pivotal trials [2–4] and observational studies [13, 14] of triple therapies mainly used occurrence of exacerbations during 1-year of follow-up as a primary endpoint. Pneumonia and cardiovascular events are also safety concerns for ICS and bronchodilators, respectively [15, 16]. Therefore, our effectiveness outcomes included severe and moderate exacerbations and the safety outcomes were pneumonia and composite cardiovascular events, which were determined based on the first corresponding episode within one year after the index date. We ascertained severe exacerbations using inpatient diagnosis codes in the primary position and ascertained moderate exacerbations according to an outpatient prescription of oral corticosteroids for 5–14 days. The pneumonia outcome was defined with inpatient diagnosis codes in any positions. The composite cardiovascular events of acute myocardial infarction, unstable angina, congestive heart failure, cardiac dysrhythmia, or ischemic stroke were determined using inpatient diagnosis codes in the primary position. The positive predictive values of these claims-based algorithms were 86% for severe exacerbations [20], 73% for moderate exacerbations [21], 88% for pneumonia [22], and 80–100% for each component of the composite cardiovascular events [23–28]. Supplementary Table 2 outlines the detailed outcome definitions.

The primary follow-up scheme was based on an on-treatment approach, which followed patients from the first day after the index date to the earliest of each outcome occurrence, study treatment discontinuation or change, death, end of data (2022/12/31), or the 365st day after the index date. We defined study treatment discontinuation with a 60-day grace period. For fluticasone/umeclidinium/vilanterol initiators, study treatment change was defined as a dispensing of beclometasone/glycopyrrolate/formoterol. For beclometasone/glycopyrrolate/formoterol initiators, study treatment change was defined as a dispensing of fluticasone/umeclidinium/vilanterol.

### Covariate measurement

We accounted for > 100 baseline covariates that were potentially associated with the exposure (use of triple therapies) and the risk of study outcomes (exacerbations, pneumonia, and cardiovascular events), including (1) age on the index date, (2) sex, (3) COPD duration, (4) general disease burden (Charlson Comorbidity Index [29] and Claim-based Frailty Index [30]), (5) comorbidities (e.g., respiratory and cardiovascular disease), (6) concomitant medication use (e.g., non-study inhaled medications and cardiovascular medications), and (7) healthcare services (e.g., pneumococcal or influenza vaccination; outpatient visits, emergency department visits, or hospital admissions; and cardiovascular systems related examinations). These pre-defined claims-based covariates were defined using corresponding healthcare data extracted within 365 days preceding the index date (except COPD duration, which was measured from the earliest recorded date of COPD diagnosis, looking back until 2014/01/01, to the index date). Supplementary Table 3 to Supplementary Table 5 show detailed covariate information.

### Statistical analysis

Considering the above pre-defined claims-based covariates, we estimated baseline propensity scores with multivariable logistic regression models. To mitigate potential unmeasured confounding, we also enriched propensity score estimation through a data-driven approach. Specifically, we used the high-dimensional propensity score algorithm, an automated algorithm that identifies and prioritizes empirical claims-based covariates that were simultaneously associated with the exposure and the risk of study outcomes [31, 32]. These covariates may serve proxies of unmeasured confounders. We selected 200 empirical covariates and combined all the empirical and pre-defined covariates to estimate high-dimensional propensity scores.

As fluticasone/umeclidinium/vilanterol was reimbursed earlier than beclometasone/glycopyrrolate/formoterol in Taiwan, it was expected that there were more fluticasone/umeclidinium/vilanterol initiators. Therefore, we employed a 5:1 variable-ratio matching that allowed up to five fluticasone/umeclidinium/vilanterol initiators being matched to one beclometasone/glycopyrrolate/formoterol initiator via a nearest-neighbor algorithm (no replacement and a matching caliper of 0.025 on the propensity score or high-dimensional propensity score scale) [33]. Standardized mean differences for individual covariates were calculated to examine their distributions before and after propensity score or high-dimensional propensity score matching. An absolute value of < 0.1 was considered a comparable distribution between treatment groups [34].

We estimated the incidence rates and 95% CIs of each outcome with a Poisson distribution and plotted corresponding cumulative incidence curves over time by treatment group. We also estimated HRs and 95% CIs comparing fluticasone/umeclidinium/vilanterol to beclometasone/glycopyrrolate/formoterol under Cox proportional hazards models. The above analyses were implemented without and with propensity score or high-dimensional propensity score matching, accounting for different matching ratios [33].

### Sensitivity analyses

We carried out the following pre-specified sensitivity analyses to evaluate if the results change materially. First, we attempted an intention-to-treat approach, which followed patients up to 365 days irrespective of study treatment discontinuation or change. Second, we executed the Fine‐Gray subdistribution hazard model to estimate subdistribution HRs, which addressed the potential influence of informative censoring due to death for any reasons [35, 36]. Third, we simultaneously adjusted for the aforementioned pre-defined claims-based covariates and 11 meaningful clinical measures collected from two external nationwide databases—the Taiwan National Health Insurance Laboratory Database and the Taiwan COPD pay-for-performance Database [37, 38]. These baseline measures included laboratory examinations, lung function test and respiratory symptoms, blood pressure, and health behavior. We performed multiple imputation to address the issue that only some of the patients had some of these measures [39].

## Subgroup analyses

To evaluate if the effectiveness and safety profiles varied by important patient characteristic and prior dual maintenance medication use, we conducted several subgroup analyses by Charlson Comorbidity Index, Claim-based Frailty Index, history of hospitalized COPD exacerbations, and previous LABA/ICS or LABA/LAMA use. To assess if the associations differed with longer treatment duration, we also separately examined HRs from the index date to 90 days for all eligible initiators and HRs from 91 to 365 days for patients treated for ≥ 90 days. We re-estimated the high-dimensional propensity scores and re-matched patients within each patient subgroup [40].

The present study was part of our series of research which sought to assess the effectiveness and safety of inhaled combination maintenance medications reimbursed in Taiwan. The reporting of the study complied with the Strengthening the Reporting of Observational Studies in Epidemiology (STROBE) statement. The cohort designs and statistical approaches adhered to the principles of pharmacoepidemiologic studies and were also described in our previous work [41].

### Ethics approval

The National Yang-Ming Chiao Tung University Research Ethics Committee approved the study on 2023/05/30 (ID: NYCU112102AE). Informed consent was waived given the retrospective nature of the study and the analysis of anonymous data.

## Results

### Baseline characteristics

There were 14,278 eligible patients (Supplementary Fig. 1). Fluticasone/umeclidinium/vilanterol initiators (n = 9750) were slightly younger, were more likely to be male, and had shorter COPD duration than beclometasone/glycopyrrolate/formoterol initiators (n = 4528). Fluticasone/umeclidinium/vilanterol initiators also had a lower general disease burden, fewer individual comorbidities, fewer medication use, and less frequent access to healthcare services. After propensity score matching and high-dimensional propensity score matching, 13,424 patients (94% of the original cohort) and 12,971 patients (90% of the original cohort) were included, respectively. All the pre-defined claims-based covariates achieved balance between treatment groups. See Table 1, Supplementary Table 6, and Supplementary Fig. 2.

**Table 1:** Select baseline characteristics among the eligible cohort

	Before matching (n = 14,278)			After propensity score matching (n = 13,424)			After high-dimensional propensity score matching (n = 12,971)		
	Fluticasone/umeclidinium/vilanterol	Beclometasone/glycopyrronium/formoterol	Standardized mean difference	Fluticasone/umeclidinium/vilanterol	Beclometasone/glycopyrronium/formoterol	Standardized mean difference	Fluticasone/umeclidinium/vilanterol	Beclometasone/glycopyrronium/formoterol	Standardized mean difference
	(n = 9750)	(n = 4528)		(n = 9489)	(n = 3935)		(n = 9171)	(n = 3800)	
				(n = 3935^a^)	(n = 3935^a^)		(n = 3800^a^)	(n = 3800^a^)	
Demographics and COPD duration									
Age, years, mean (SD)	70.15 (10.15)	71.96 (10.98)	− 0.171	71.06 (10.72)	70.92 (10.64)	0.013	71.00 (10.65)	71.00 (10.66)	< 0.001
Male, n (%)	8733 (89.57)	3787 (83.64)	0.175	3391 (86.18)	3383 (85.97)	0.006	3264 (85.89)	3260 (85.79)	0.003
COPD duration, days, mean (SD)^b^	1649 (934.42)	1769 (1045.59)	− 0.121	1768 (927.81)	1768 (1033.79)	0.001	1752 (947.05)	1768 (1032.84)	− 0.017
General disease burden, mean (SD)									
Charlson Comorbidity Index	3.10 (2.42)	3.44 (2.58)	− 0.135	3.25 (2.50)	3.21 (2.43)	0.016	3.21 (2.47)	3.18 (2.41)	0.010
Claim-based Frailty Index	0.14 (0.13)	0.19 (0.17)	− 0.306	0.16 (0.15)	0.16 (0.14)	0.027	0.16 (0.14)	0.16 (0.14)	0.015
Comorbidities, n (%)									
Pneumonia	1329 (13.63)	902 (19.92)	− 0.169	642 (16.32)	624 (15.86)	0.012	561 (14.76)	580 (15.26)	− 0.014
Influenza	286 (2.93)	94 (2.08)	0.055	84 (2.13)	87 (2.21)	− 0.005	93 (2.45)	85 (2.24)	0.014
Acute bronchitis	3766 (38.63)	1942 (42.89)	− 0.087	1535 (39.01)	1541 (39.16)	− 0.003	1464 (38.53)	1450 (38.16)	0.008
COVID-19	284 (2.91)	341 (7.53)	− 0.209	192 (4.88)	197 (5.01)	− 0.006	186 (4.89)	194 (5.11)	− 0.010
Post COVID condition	27 (0.28)	28 (0.62)	− 0.051	17 (0.43)	19 (0.48)	− 0.008	13 (0.34)	16 (0.42)	− 0.013
Hypertension	5700 (58.46)	2802 (61.88)	− 0.070	2350 (59.72)	2346 (59.62)	0.002	2284 (60.11)	2243 (59.03)	0.022
Ischemic heart disease or angina	2755 (28.26)	1409 (31.12)	− 0.063	1200 (30.50)	1172 (29.78)	0.016	1140 (30)	1132 (29.79)	0.005
Myocardial infarction	314 (3.22)	187 (4.13)	− 0.048	150 (3.81)	143 (3.63)	0.009	133 (3.5)	129 (3.39)	0.006
Cardiac dysrhythmia	1583 (16.24)	895 (19.77)	− 0.092	727 (18.48)	711 (18.07)	0.011	95 (2.5)	92 (2.42)	0.005
Congestive heart failure	1679 (17.22)	996 (22.00)	− 0.121	766 (19.47)	764 (19.42)	0.001	667 (17.55)	673 (17.71)	− 0.004
Cerebrovascular disease	1212 (12.43)	924 (20.41)	− 0.217	644 (16.37)	622 (15.81)	0.015	699 (18.39)	708 (18.63)	− 0.006
Peripheral vascular disease	295 (3.03)	134 (2.96)	0.004	119 (3.02)	106 (2.69)	0.020	599 (15.76)	601 (15.82)	− 0.001
Diabetes mellitus	2670 (27.38)	1398 (30.87)	− 0.077	1115 (28.34)	1120 (28.46)	− 0.003	282 (7.42)	292 (7.68)	− 0.010
Hyperlipidemia	3629 (37.22)	1651 (36.46)	0.016	1426 (36.24)	1428 (36.29)	− 0.001	74 (1.95)	76 (2)	− 0.004
Medications, n (%)									
Inhaled short-acting bronchodilators	5985 (61.38)	2988 (65.99)	− 0.096	2525 (64.17)	2505 (63.66)	0.011	2377 (62.55)	2373 (62.45)	0.002
LABA	100 (1.03)	52 (1.15)	− 0.012	38 (0.97)	48 (1.22)	− 0.024	44 (1.16)	46 (1.21)	− 0.005
LAMA	2748 (28.18)	1374 (30.34)	− 0.047	1240 (31.51)	1209 (30.72)	0.017	1167 (30.71)	1167 (30.71)	NA
LABA/ICS FDC	3547 (36.38)	1985 (43.84)	− 0.153	1709 (43.43)	1725 (43.84)	− 0.008	1641 (43.18)	1648 (43.37)	− 0.004
LABA/LAMA FDC	5889 (60.40)	1895 (41.85)	0.378	1830 (46.51)	1794 (45.59)	0.018	1744 (45.89)	1725 (45.39)	0.010
ICS	1094 (11.22)	509 (11.24)	− 0.001	437 (11.11)	412 (10.47)	0.020	421 (11.08)	405 (10.66)	0.014
Systemic bronchodilators	7302 (74.89)	3471 (76.66)	− 0.041	3001 (76.26)	3027 (76.93)	− 0.016	2942 (77.42)	2924 (76.95)	0.011
Systemic corticosteroids	6393 (65.57)	3227 (71.27)	− 0.123	2713 (68.95)	2723 (69.20)	− 0.005	2586 (68.05)	2603 (68.50)	− 0.010
Oral antibiotics commonly used for COPD exacerbations	6010 (61.64)	3045 (67.25)	− 0.117	2536 (64.45)	2542 (64.60)	− 0.003	2428 (63.89)	2417 (63.61)	0.006
ACEIs or ARBs	4346 (44.57)	2154 (47.57)	− 0.060	1813 (46.07)	1831 (46.53)	− 0.009	1758 (46.26)	1759 (46.29)	− 0.001
Selective β blockers	2431 (24.93)	1359 (30.01)	− 0.114	1102 (28.01)	1094 (27.80)	0.005	1031 (27.13)	1020 (26.84)	0.007
Non-selective β blockers	1107 (11.35)	546 (12.06)	− 0.022	458 (11.64)	438 (11.13)	0.016	429 (11.29)	419 (11.03)	0.008
DHP CCBs	2822 (28.94)	1440 (31.80)	− 0.062	1159 (29.45)	1160 (29.48)	− 0.001	1113 (29.29)	1097 (28.87)	0.009
Non-DHP CCBs	1464 (15.02)	775 (17.12)	− 0.057	616 (15.65)	640 (16.26)	− 0.017	613 (16.13)	611 (16.08)	0.001
Diuretics	2833 (29.06)	1750 (38.65)	− 0.204	1358 (34.51)	1329 (33.77)	0.016	1263 (33.24)	1238 (32.58)	0.014
Other anti-hypertensive agents	1133 (11.62)	561 (12.39)	− 0.024	455 (11.56)	457 (11.61)	− 0.002	454 (11.95)	428 (11.26)	0.021
Nitrates	1788 (18.34)	894 (19.74)	− 0.036	726 (18.45)	747 (18.98)	− 0.014	713 (18.76)	708 (18.63)	0.003
Anti-arrhythmic agents	951 (9.75)	594 (13.12)	− 0.106	446 (11.33)	436 (11.08)	0.008	407 (10.71)	403 (10.61)	0.003
Digoxin	259 (2.66)	182 (4.02)	− 0.076	131 (3.33)	123 (3.13)	0.012	126 (3.32)	121 (3.18)	0.007
Aspirin	2669 (27.37)	1384 (30.57)	− 0.070	1147 (29.15)	1139 (28.95)	0.004	1075 (28.29)	1085 (28.55)	− 0.006
Clopidogrel	1106 (11.34)	618 (13.65)	− 0.070	489 (12.43)	490 (12.45)	− 0.001	476 (12.53)	467 (12.29)	0.007
Warfarin	115 (1.18)	79 (1.74)	− 0.047	53 (1.35)	58 (1.47)	− 0.011	51 (1.34)	59 (1.55)	− 0.018
New oral anticoagulants	677 (6.94)	380 (8.39)	− 0.054	301 (7.65)	297 (7.55)	0.004	293 (7.71)	291 (7.66)	0.002
Statins	3167 (32.48)	1457 (32.18)	0.007	1274 (32.38)	1251 (31.79)	0.013	1225 (32.24)	1226 (32.26)	− 0.001
Fibrates	326 (3.34)	134 (2.96)	0.022	122 (3.10)	114 (2.90)	0.012	115 (3.03)	111 (2.92)	0.006
Insulin	883 (9.06)	733 (16.19)	− 0.216	475 (12.07)	480 (12.20)	− 0.004	446 (11.74)	439 (11.55)	0.006
Metformin	1730 (17.74)	824 (18.20)	− 0.012	684 (17.38)	690 (17.53)	− 0.004	661 (17.39)	664 (17.47)	− 0.002
Sulfonylureas	875 (8.97)	402 (8.88)	0.003	345 (8.77)	332 (8.44)	0.012	343 (9.03)	329 (8.66)	0.013
Glinides	192 (1.97)	172 (3.80)	− 0.109	120 (3.05)	95 (2.41)	0.039	105 (2.76)	97 (2.55)	0.013
Thiazolidinedione	290 (2.97)	118 (2.61)	0.022	98 (2.49)	105 (2.67)	− 0.011	100 (2.63)	103 (2.71)	− 0.005
Alpha-glucosidase inhibitors	224 (2.30)	108 (2.39)	− 0.006	89 (2.26)	85 (2.16)	0.007	82 (2.16)	80 (2.11)	0.004
Dipeptidyl peptidase-4 inhibitors	1180 (12.10)	670 (14.80)	− 0.079	512 (13.01)	503 (12.78)	0.007	500 (13.16)	499 (13.13)	0.001
Sodium-glucose cotransporter 2 Inhibitors	385 (3.95)	245 (5.41)	− 0.069	188 (4.78)	189 (4.80)	− 0.001	180 (4.74)	187 (4.92)	− 0.009
Glucagon-like peptide-1 receptor agonists	39 (0.40)	13 (0.29)	0.019	13 (0.33)	13 (0.33)	NA	13 (0.34)	13 (0.34)	NA
Healthcare services									
Vaccination, n (%)									
Pneumococcal or influenza vaccination	5763 (59.11)	3035 (67.03)	− 0.165	2528 (64.24)	2524 (64.14)	0.002	2428 (63.89)	2414 (63.53)	0.008
Resource utilization, mean (SD)									
No. of any outpatient visits	41.81 (26.32)	40.60 (26.02)	0.046	40.78 (25.78)	40.52 (26.34)	0.010	40.8 (25.79)	40.4 (26.33)	0.015
No. of outpatient visits due to COPD	11.50 (8.95)	10.73 (9.56)	0.083	10.94 (8.89)	10.92 (9.17)	0.002	10.79 (8.69)	10.92 (9.38)	− 0.014
No. of outpatient visits due to pneumonia	0.46 (2.31)	0.52 (2.31)	− 0.025	0.51 (2.49)	0.49 (2.29)	0.007	0.48 (2.35)	0.47 (2.16)	0.005
No. of outpatient visits due to cardiovascular disease^c^	12.06 (12.50)	13.03 (13.34)	− 0.075	12.43 (12.55)	12.29 (13.01)	0.011	12.41 (12.46)	12.21 (12.88)	0.016
No. of emergency department visits	1.08 (2.15)	1.71 (3.07)	− 0.238	1.35 (2.63)	1.37 (2.37)	− 0.008	1.34 (2.67)	1.3 (2.23)	0.016
No. of emergency department visits due to COPD	0.32 (1.04)	0.51 (1.65)	− 0.135	0.41 (1.18)	0.42 (1.13)	− 0.006	0.41 (1.25)	0.38 (1.08)	0.024
No. of emergency department visits due to pneumonia	0.05 (0.27)	0.07 (0.34)	− 0.069	0.05 (0.28)	0.05 (0.28)	< 0.001	0.05 (0.28)	0.05 (0.29)	− 0.007
No. of emergency department visits due to cardiovascular disease^c^	0.22 (0.79)	0.36 (0.97)	− 0.158	0.28 (0.93)	0.27 (0.83)	0.003	0.27 (0.88)	0.26 (0.8)	0.009
No. of any hospitalizations	0.76 (1.46)	1.07 (1.77)	− 0.191	0.89 (1.61)	0.89 (1.63)	0.003	0.85 (1.57)	0.83 (1.59)	0.011
No. of hospitalizations due to COPD	0.49 (1.11)	0.68 (1.35)	− 0.152	0.57 (1.19)	0.57 (1.21)	0.004	0.54 (1.2)	0.53 (1.19)	0.012
No. of hospitalizations due to pneumonia	0.05 (0.27)	0.13 (0.48)	− 0.201	0.08 (0.34)	0.07 (0.30)	0.040	0.07 (0.3)	0.07 (0.3)	0.002
No. of hospitalizations due to cardiovascular disease^c^	0.51 (1.18)	0.79 (1.55)	− 0.209	0.62 (1.34)	0.62 (1.39)	− 0.005	0.61 (1.36)	0.57 (1.34)	0.026
Spirometry test and cardiovascular systems related examinations, n (%)									
Spirometry test	7013 (71.93)	2481 (54.79)	0.361	2401 (61.02)	2399 (60.97)	0.001	2361 (62.13)	2331 (61.34)	0.016
Echochardiography	3119 (31.99)	1689 (37.30)	− 0.112	1381 (35.10)	1363 (34.64)	0.010	1297 (34.13)	1286 (33.84)	0.006
24-h ECG examination	590 (6.05)	283 (6.25)	− 0.008	239 (6.07)	222 (5.64)	0.018	216 (5.68)	213 (5.61)	0.003
BNP, proBNP, or NT-proBNP test	2558 (26.24)	1607 (35.49)	− 0.201	1195 (30.37)	1226 (31.16)	− 0.017	1135 (29.87)	1114 (29.32)	0.012

*ACEI* angiotensin converting enzyme inhibitor, *ARB* angiotensin receptor blocker, *BNP* B-type natriuretic peptide, *CCB* calcium channel blocker, *COPD* chronic obstructive pulmonary disease, *COVID* coronavirus disease, *DHP* dihydropyridine, *ECG* electrocardiography, *FDC* fixed-dose combinations, *ICS* inhaled corticosteroids, *LABA* long-acting β_2_ agonists, *LAMA* long-acting muscarinic antagonists, *NA* not applicable, *NT* n-terminal, *SD* standard deviation

^a^One randomly sampled fluticasone/umeclidinium/vilanterol initiator versus one beclometasone/glycopyrronium/formoterol initiator in each matched subset

^b^COPD duration was defined as the duration from the first recorded date of COPD diagnosis (looking back until 2014/01/01) to the index date

^c^Cardiovascular disease include hypertension, ischemic heart disease or angina, myocardial infarction, coronary revascularization, cardiac dysrhythmia, congestive heart failure, cerebrovascular disease, ischemic stroke, hemorrhagic stroke, transient ischemic attack, peripheral vascular disease, diabetes mellitus, and hyperlipidemia

### Risk of study outcomes associated with fluticasone/umeclidinium/vilanterol versus beclometasone/glycopyrrolate/formoterol

Before matching, the mean follow-up duration was nearly 220 days. The crude incidence rates of individual outcomes were lower in the fluticasone/umeclidinium/vilanterol initiators than in the beclometasone/glycopyrrolate/formoterol initiators (Table 2), with a crude HR of 0.67 (0.59–0.77) for severe exacerbations, 0.80 (0.74–0.86) for moderate exacerbations, 0.52 (0.45–0.61) for pneumonia, and 0.71 (0.55–0.95) for composite cardiovascular events (Table 3).

**Table 2:** Follow-up and incidence rate of study outcomes among the eligible cohort

	Before matching		After propensity score matching		After high-dimensional propensity score matching	
	Fluticasone/umeclidinium/vilanterol	Beclometasone/glycopyrronium/formoterol	Fluticasone/umeclidinium/vilanterol	Beclometasone/glycopyrronium/formoterol	Fluticasone/umeclidinium/vilanterol	Beclometasone/glycopyrronium/formoterol
*Severe exacerbations*						
No. of patients (no. of events)	9750 (604)	4528 (356)	9489 (585)	3,935 (312)	9,171 (558)	3,800 (278)
Follow-up days, mean (SD)	233.32 (127.93)	192.21 (128.51)	232.75 (128.12)	196.90 (128.44)	232.35 (127.89)	199.03 (128.34)
Incidence rate per 1,000 person-years (95% CI)^a^	96.98 (89.54, 105.03)	149.40 (134.66, 165.76)	114.94 (102.12, 129.36)	147.08 (131.63, 164.34)	108.18 (95.58, 122.44)	134.26 (119.37, 151.00)
*Moderate exacerbations*						
No. of patients (no. of events)	9750 (2094)	4528 (1041)	9489 (2040)	3,935 (933)	9,230 (1,945)	3,822 (897)
Follow-up days, mean (SD)	207.19 (131.40)	168.78 (126.91)	206.51 (131.48)	172.43 (127.74)	206.98 (131.41)	173.79 (127.77)
Incidence rate per 1,000 person-years (95% CI)^a^	378.61 (362.74, 395.18)	497.53 (468.21, 528.69)	392.56 (366.83, 420.09)	502.24 (471.03, 535.53)	381.39 (355.76, 408.87)	493.24 (462.00, 526.60)
*Pneumonia*						
No. of patients (no. of events)	9750 (387)	4528 (291)	9489 (377)	3,935 (210)	9,311 (363)	3,815 (179)
Follow-up days, mean (SD)	238.11 (126.94)	197.05 (127.44)	237.55 (127.14)	203.23 (128.37)	238.17 (127.20)	204.96 (127.95)
Incidence rate per 1,000 person-years (95% CI)^a^	60.89 (55.11, 67.26)	119.12 (106.19, 133.63)	73.20 (63.23, 84.74)	95.91 (83.78, 109.80)	69.61 (59.78, 81.06)	83.61 (72.22, 96.81)
*Composite cardiovascular events*						
No. of patients (no. of events)	9750 (138)	4528 (77)	9489 (135)	3,935 (60)	9,355 (126)	3,816 (52)
Follow-up days, mean (SD)	240.49 (126.32)	201.58 (127.83)	239.94 (126.51)	206.63 (126.75)	239.92 (126.47)	208.25 (127.38)
Incidence rate per 1,000 person-years (95% CI)^a^	21.50 (18.19, 25.40)	30.81 (24.64, 38.52)	24.42 (18.99, 31.41)	26.95 (20.93, 34.713)	22.55 (17.29, 29.41)	23.90 (18.21, 31.37)

*CI* confidence interval, *SD* standard deviation

^a^The incidence rate after propensity score or high-dimensional propensity score matching was weighted by the inverse of the matching ratio

**Table 3:** HR (95% CI) of study outcomes comparing fluticasone/umeclidinium/vilanterol with beclometasone/glycopyrrolate/formoterol

	Before matching	After propensity score matching	After high-dimensional propensity score matching
Severe exacerbations	0.67 (0.59, 0.77)	0.79 (0.68, 0.91)	0.80 (0.69, 0.93)
Moderate exacerbations	0.80 (0.74, 0.86)	0.81 (0.75, 0.88)	0.80 (0.74, 0.87)
Pneumonia	0.52 (0.45, 0.61)	0.78 (0.65, 0.93)	0.85 (0.70, 1.02)
Composite cardiovascular events	0.72 (0.55, 0.95)	0.94 (0.69, 1.29)	0.96 (0.69, 1.35)

*CI* confidence interval, *HR* hazard ratio

The HR after propensity score or high-dimensional propensity score matching was stratified on the matching ratio

After propensity score and high-dimensional propensity score matching, differences in incidence rates between treatment groups reduced (Table 2). The HR after high-dimensional propensity score matching suggested a lower risk of severe and moderate exacerbations (0.80 [0.69–0.93] and 0.80 [0.74–0.87], respectively) and a marginally non-significant decreased risk of pneumonia (0.85 [0.70–1.02]) associated with fluticasone/umeclidinium/vilanterol. However, both treatments showed a similar risk of composite cardiovascular events (0.96 [0.69–1.35]) (Table 3). The cumulative incidence plots corresponded to above outcome estimates (Supplementary Fig. 3).

### Findings of sensitivity analyses

**Fig. 1:**
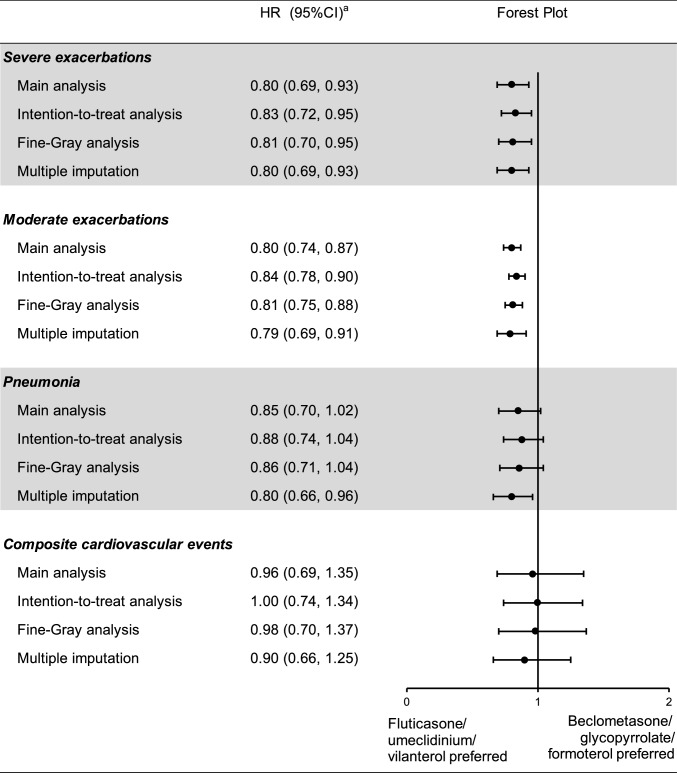
HR (95% CI)^a^ of sensitivity analyses comparing fluticasone/umeclidinium/vilanterol with beclometasone/glycopyrrolate/formoterol, after high-dimensional propensity score matching or after propensity score matching with multiple imputation. CI, confidence interval; HR, hazard ratio. ^a^The HR after high-dimensional propensity score or propensity score matching was stratified on the matching ratio

Compared to the on-treatment approach, the intention-to-treat analysis obtained a longer follow-up duration (nearly 277 days before matching). The HR after high-dimensional propensity score matching was 0.83 (0.72–0.95) for severe exacerbations, 0.84 (0.78–0.90) for moderate exacerbations, 0.88 (0.74–1.04) for pneumonia, and 1.00 (0.74–1.34) for composite cardiovascular events (Supplementary Table 7 to Supplementary Table 9). The Fine-Gray subdistribution HR did not vary apparently compared to that estimated with the conventional Cox model (Supplementary Table 8 and Supplementary Table 9). Approximately 91% of initiators had at least one clinical measure collected from the NHI Laboratory Database or the COPD P4P Database (Supplementary Table 10). When the above pre-defined claims-based covariates and clinical measures were incorporated into the propensity score matching using a multiple imputation technique, the distribution of the measures between treatment groups became similar (Supplementary Table 11). The risk estimates were in line with those calculated in the main analysis (Supplementary Table 8 and Supplementary Table 9). Figure 1 visualizes the results of sensitivity analyses.

### Findings of subgroup analyses

**Fig. 2:**
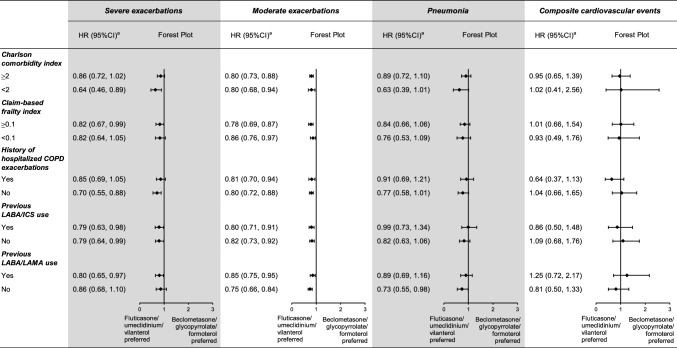
HR (95% CI)^a^ of subgroup analyses comparing fluticasone/umeclidinium/vilanterol with beclometasone/glycopyrrolate/formoterol by important patient characteristic and prior dual maintenance medication use, after high-dimensional propensity score matching. CI, confidence interval; COPD, chronic obstructive pulmonary disease; HR, hazard ratio; ICS, inhaled corticosteroids; LABA, long-acting β_2_ agonists; LAMA, long-acting muscarinic antagonists. ^a^The HR after high-dimensional propensity score o matching was stratified on the matching ratio

Overall, the results did not change materially by important patient characteristic and prior dual maintenance medication use (Fig. 2, Supplementary Table 12, and Supplementary Table 13). However, the treatment duration response analysis revealed that the effectiveness and safety profiles of the two treatments appeared more comparable among patients who had been treated for ≥ 90 days (approximately 73% of the eligible initiators). The HR after high-dimensional propensity score matching from 91 to 365 days was 0.98 (0.78–1.23) for severe exacerbations, 0.93 (0.72–1.20) for pneumonia, and 1.07 (0.64–1.77) for composite cardiovascular events. Nevertheless, fluticasone/umeclidinium/vilanterol remained having a lower risk of moderate exacerbations (0.86 [0.74–0.98]). (Table 4, Supplementary Table 12, and Supplementary Table 13).

**Table 4:** HR (95% CI) of subgroup analyses comparing fluticasone/umeclidinium/vilanterol with beclometasone/glycopyrrolate/formoterol by treatment duration, after high-dimensional propensity score matching

	Eligible initiators (n = 12,971)	Patients treated for ≥ 90 days (n = 9472)
	From the index date to 90 days	From 91 to 365 days
Severe exacerbations	0.68 (0.56, 0.84)	0.98 (0.78, 1.23)
Moderate exacerbations	0.78 (0.70, 0.87)	0.86 (0.74, 0.98)
Pneumonia	0.80 (0.60, 1.06)	0.93 (0.72, 1.20)
Composite cardiovascular events	0.89 (0.56, 1.42)	1.07 (0.64, 1.77)

*CI* confidence interval, *HR* hazard ratio

The HR after high-dimensional propensity score matching was stratified on the matching ratio

## Discussion

In this population-based cohort study with approximately 13,000 initiators of single-inhaler triple therapies, fluticasone/umeclidinium/vilanterol (a dry powder inhaler) was associated with a decreased risk of exacerbations and a marginally non-significant lower risk of pneumonia, while exhibiting a similar risk of composite cardiovascular events compared to beclometasone/glycopyrrolate/formoterol (a metered dose inhaler). The findings remained robust in sensitivity analyses that addressed potential informative censoring due to treatment discontinuation or change or death, and potential confounding effects from clinical measures. The results were also consistent across patient subgroups by key characteristic and prior dual maintenance medication use. Notably, the benefits and risks of both treatments appeared more comparable among patients who had maintained their therapies for ≥90 days.

### Comparative studies for inhaled triple therapies in the real-world settings

Real-world studies directly comparing inhaled triple therapies remain scarce, with most focusing on fluticasone-based versus budesonide-based treatments and investigating respiratory outcomes. An early U.K. cohort study conducted by Suissa et al. reported that the fluticasone-based triple therapy group had a higher risk of exacerbations and pneumonia than the budesonide-based triple therapy group [42]. Fluticasone is hypothesized to have lower aqueous solubility than budesonide. This may affect dissolution and uptake in the respiratory tract, thereby reducing drug effectiveness, impairing macrophage function, and increasing the risk of exacerbations and pneumonia [8, 9]. Notably, this study assessed patients who used triple therapies through separate inhalers and analyzed data on fluticasone propionate. Tiotropium was the predominant LAMA, while salmeterol and formoterol were the main LABA. In contrast, two U.S. cohort studies conducted by Mannino et al. [13] and Feldman et al. [14] compared single-inhaler therapies and found that fluticasone/umeclidinium/vilanterol may have a lower risk of exacerbations than budesonide/glycopyrrolate/formoterol. Feldman et al. also showed both treatments had a similar risk of pneumonia [14]. Both U.S. studies examined fluticasone furoate, which has a longer duration of anti-inflammatory effect than fluticasone propionate [8, 9]. Importantly, none of these three studies assessed cardiovascular outcomes.

To our knowledge, the present cohort study is the first study to evaluate the effectiveness and safety of fluticasone/umeclidinium/vilanterol and beclometasone/glycopyrrolate/formoterol, providing real-world evidence to guide treatment selection in Europe and Asia where beclometasone/glycopyrrolate/formoterol is available for COPD management. Besides exacerbations and pneumonia, we examined a broad spectrum of cardiovascular outcomes. The comparable cardiovascular safety profiles between the two treatments suggest that treatment decisions can be made with minimal concerns about cardiovascular risks. Our results indicate that fluticasone/umeclidinium/vilanterol may be a preferred initial option, consistent with the findings of Mannino et al. and Feldman et al. [13, 14] and aligned with ongoing efforts to reduce greenhouse gas emissions from metered dose inhalers [43, 44].

### Insight into more comparable benefits and risks of the two inhaled triple therapies among patients who had maintained the medications for ≥ 90 days

On the other hand, fluticasone/umeclidinium/vilanterol delivered via a dry powder inhaler may not be clinically suitable for all patients. Specifically, different inhaler devices require distinct techniques [45]. While patients using dry powder inhalers need deep inspiration, patients using metered dose inhalers should be able to slow down their breathing. Our results showed more comparable benefits and risks of the two treatments among patients treated for ≥ 90 days, supporting the argument that beclometasone/glycopyrrolate/formoterol delivered via a metered dose inhaler may be a viable alternative as long as patients can maintain their treatments for some time. The findings offer flexibility for individualized treatments, particularly for patients who experience difficulties with dry powder inhalers or who prefer metered dose inhalers.

### Strengths and limitations

A key strength of this population-based cohort study is the comparison of respiratory and cardiovascular outcomes between fluticasone-based and beclometasone-based triple therapies in an Asian population and across diverse patient subgroups. By extending previous findings from Western studies that compared fluticasone-based and budesonide-based treatments with a focus on respiratory outcomes, our study provides complementary real-world evidence across geographic areas and clinical contexts. In terms of data source, leveraging the nationwide Taiwanese database enabled access to comprehensive information on single-inhaler therapies, clinical outcomes, and a wide range of covariates. Furthermore, the consistent results across main and sensitivity analyses corroborate the robustness of our findings.

Our study has several limitations. First, to minimize potential misdiagnosis and treatment effect heterogeneity between COPD and asthma, we excluded patients with a history of asthma and cannot apply our findings to this population accordingly. Second, we adopted an on-treatment follow-up approach based on pharmacy claims data to address medication adherence and its potential influence on outcome estimates. However, fluticasone/umeclidinium/vilanterol is administered once daily, whereas beclometasone/glycopyrrolate/formoterol is administered twice daily. Patients who used beclometasone/glycopyrrolate/formoterol may tend to omit doses, leading to lower effectiveness. We also could not evaluate their inhaler techniques. Future studies with detailed adherence and technique data are warranted to clarify whether the observed differences between treatment groups reflect these factors or the distinct physicochemical and pharmacokinetic profiles of the individual drug moieties. Third, budesonide/glycopyrrolate/formoterol was not marketed in Taiwan during the study period, precluding comparison across all three single-inhaler triple therapies. Finally, patients who used fluticasone/umeclidinium/vilanterol tended to be healthier than patients who used beclometasone/glycopyrrolate/formoterol (see Table 1), which may partly explain the benefits associated with fluticasone/umeclidinium/vilanterol. Nevertheless, besides adjusting for pre-defined claims-based covariates, we considered empirical claims-based covariates and clinical measures in the analysis to mitigate potential unmeasured confounding.

## Conclusion

This population-based Asian cohort study suggests that fluticasone/umeclidinium/vilanterol may be a preferred initial treatment option over beclometasone/glycopyrrolate/formoterol. While among patients who are able to maintain their therapies for ≥ 90 days, both treatments may demonstrate more comparable effectiveness and safety profiles.

## Supplementary Information

Below is the link to the electronic supplementary material.

Supplementary file1 (DOCX 475 KB)

## Data Availability

The data used in the current study were obtained from the Applied Health Research Data Integration Service from the National Health Insurance Administration, Taiwan, which are not publicly available given the data protection policy. However, the authors are willing to have further discussion if there are any questions.
