# Supplementary material for: Comparative effectiveness and safety of fluticasone-based versus beclometasone-based single-inhaler triple therapies in patients with chronic obstructive pulmonary disease: a population-based cohort study
Source: Int J Clin Pharm. 2025 Nov 7;48(2):607–22. doi: 10.1007/s11096-025-02037-4 (PMC12992448; doi:10.1007/s11096-025-02037-4)
Supplement: Supplementary file 1 — Supplementary file1 (DOCX 475 KB) [file 11096_2025_2037_MOESM1_ESM.docx]

**Supplementary Information**

**Comparative effectiveness and safety of fluticasone-based versus beclometasone-based single-inhaler triple therapies in patients with chronic obstructive pulmonary disease: a population-based cohort study**

| **Supplementary Method** |  |
| --- | --- |
| **Identification of a study cohort who initiated single-inhaler triple therapies** | P3 |
|  |  |
| **Supplementary Table** |  |
| **Supplementary Table 1.** Anatomical Therapeutic Chemical (ATC) classification system codes used to identify use of LABA/LAMA/ICS FDC treatments reimbursed in Taiwan between 2019-2022 | P4 |
|  |  |
| **Supplementary Table 2.** International Classification of Diseases, 10^th^ Revision, Clinical Modification (ICD-10-CM) diagnosis codes or Anatomical Therapeutic Chemical (ATC) classification system codes used to identify outcomes of interest | P5 |
|  |  |
| **Supplementary Table 3.** International Classification of Diseases, 10^th^ Revision, Clinical Modification (ICD-10-CM) diagnosis or procedure codes or Taiwan health insurance service claims codes used to identify comorbidities at baseline | P6 |
|  |  |
| **Supplementary Table 4.** Anatomical Therapeutic Chemical (ATC) classification system codes used to identify medication use at baseline | P9 |
|  |  |
| **Supplementary Table 5.** International Classification of Diseases, 10^th^ Revision, Clinical Modification (ICD-10-CM) diagnosis or procedure codes or Taiwan health insurance service claims codes used to measure healthcare services at baseline | P11 |
|  |  |
| **Supplementary Table 6.** Baseline characteristics among the eligible cohort | P13 |
|  |  |
| **Supplementary Table 7.** Follow-up, incidence rate, and HR of study outcomes among the eligible cohort based on an intention-to-treat analysis | P18 |
|  |  |
| **Supplementary Table 8.** Sensitivity analyses for severe and moderate exacerbations comparing fluticasone/umeclidinium/vilanterol with beclometasone/glycopyrrolate/formoterol, after high-dimensional propensity score matching or after propensity score matching with multiple imputation | P20 |
|  |  |
| **Supplementary Table 9.** Sensitivity analyses for pneumonia and composite cardiovascular events comparing fluticasone/umeclidinium/vilanterol with beclometasone/glycopyrrolate/formoterol, after high-dimensional propensity score matching or after propensity score matching with multiple imputation | P21 |
|  |  |
| **Supplementary Table 10.** Number (%) of patients with baseline clinical measures among the eligible cohort | P22 |
|  |  |
| **Supplementary Table 11.** Clinical measures among the imputed cohort | P23 |
|  |  |
| **Supplementary Table 12**. Subgroup analyses for severe and moderate exacerbations comparing fluticasone/umeclidinium/vilanterol with beclometasone/glycopyrrolate/formoterol by important patient characteristic, prior dual maintenance medication use, and treatment duration, after high-dimensional propensity score matching | P25 |
|  |  |
| **Supplementary Table 13**. Subgroup analyses for pneumonia and composite cardiovascular events comparing fluticasone/umeclidinium/vilanterol with beclometasone/glycopyrrolate/formoterol by important patient characteristic, prior dual maintenance medication use, and treatment duration, after high-dimensional propensity score matching | P27 |
|  |  |
| **eFigure** |  |
| **Supplementary Figure 1.** Cohort identification | P29 |
|  |  |
| **Supplementary Figure 2.** Propensity score and high-dimensional propensity score plots among the eligible cohort | P30 |
|  |  |
| **Supplementary Figure 3.** Cumulative incidence plots of study outcomes among the initiation cohort, after high-dimensional propensity score matching | P32 |
|  |  |
| **eReferences** | P34 |

**Supplementary Method**

**Identification of a study cohort who initiated single-inhaler triple therapies**

The study population included patients with chronic obstructive pulmonary disease (COPD) who initiated fluticasone/umeclidinium/vilanterol or beclometasone/glycopyrrolate/formoterol (codes in **eTable 1**) from the Taiwan NHIRD between 2019/01/01 and 2022/12/31. The index date was the date of the first dispensing of a single-inhaler triple therapy following a diagnosis of COPD. We excluded

- Patients aged less than 40 years or more than 100 years at COPD diagnosis.
- Patients with ambiguous or missing age or sex information.
- Patients who did not have interactions with the healthcare systems, defined as without any outpatient, community pharmacy, or inpatient visits within 365 days preceding the index date.
- Patients who received single-inhaler triple therapies within 365 days preceding the index date.
- Patients who simultaneously received two types of single-inhaler triple therapies or inhaled medications that contains any components of the opposite single-inhaler triple therapies on the index date.
- Patients who had a diagnosis of asthma within 365 days preceding or on the index date.
- Patients who had death records preceding the index date.
- Patients who had an index date on 2022/12/31.

See below for visualization of the cohort identification steps.

NHI, national health insurance.

**Supplementary Table 1.** Anatomical Therapeutic Chemical (ATC) classification system codes^a,b^ used to identify use of LABA/LAMA/ICS FDC treatments reimbursed in Taiwan between 2019-2022

| Medications and dosage (brand name) | ATC codes | Device | Reimbursement date | Reimbursement price (NTD) |
| --- | --- | --- | --- | --- |
| Fluticasone furoate 92μg/Umeclidinium 55μg/Vilanterol 22μg once daily (Trelegy Ellipta ^®^) | R03AL08 | Dry powder inhaler | 2019/07/01 | 1,603-1,770 |
| Beclometasone dipropionate 87μg/Glycopyrronium 9μg/Formoterol 5μg twice daily (Trimbow^®^) | R03AL09 | Metered dose inhaler | 2020/03/01 | 1,527-1,770 |

FDC, fixed-dose combination; ICS, Inhaled corticosteroids; LABA, long-acting β_2_ agonists; LAMA, long-acting muscarinic antagonists; NTD, New Taiwan dollar.

^a^Based on outpatient pharmacy dispensing claims.

^b^<https://info.nhi.gov.tw/INAE3000/INAE3000S01> Accessed on 10 January, 2025.

**Supplementary Table 2.** International Classification of Diseases, 10^th^ Revision, Clinical Modification (ICD-10-CM) diagnosis codes or Anatomical Therapeutic Chemical (ATC) classification system codes used to identify outcomes of interest

| Outcomes | Codes | PPVs |
| --- | --- | --- |
| Severe exacerbations^a^ | ICD-10-CM diagnosis codes: J41-J44 | 85.6%^d^ [1] |
| Moderate exacerbations^b^ | ATC classification system codes: H02AB, restricting to an oral form | 73.0% [2] |
| Pneumonia^c^ | ICD-10-CM diagnosis codes: J12-J17, J18.0-J18.2, A481, A37.91, A22.1, B25.0, B44.0 | 88.0%^e^ [3] |
| Composite cardiovascular events^a^ |  |  |
| Acute myocardial infarction | ICD-10-CM diagnosis codes: I21 | 100% [4] |
| Unstable angina | ICD-10-CM diagnosis codes: I20.0 | 88.9% [5] |
| Congestive heart failure | ICD-10-CM diagnosis codes: I50 | 95.9% [6] |
| Cardiac dysrhythmia | ICD-10-CM diagnosis codes: I46.9, I47.2, I49.0, R99 | 79.7%^f^ [7, 8] |
| Ischemic stroke | ICD-10-CM diagnosis codes: I63 | 99.8% [9] |

PPV, positive predicted value.

^a^Based on the primary diagnosis position in the inpatient claims.

^b^Based on outpatient pharmacy dispensing claims.

^c^Based on any diagnosis positions in the inpatient claims.

^d^The PPV was based on ICD-9-CM diagnosis codes of 491, 492, 496; mapping to ICD-10-CM codes was conducted according to clinical opinions.

^e^The PPV was based on ICD-9-CM diagnosis codes of480-486; mapping to ICD-10-CM codes was conducted according to clinical opinions.

^f^The PPV was based on ICD-9-CM diagnosis codes of 427.1, 427.4, 427.41, 427.42, 427.5, 798, 798.1, and 798.2; mapping to ICD-10-CM codes was conducted according to clinical opinions.

**Supplementary Table 3.** International Classification of Diseases, 10^th^ Revision, Clinical Modification (ICD-10-CM) diagnosis or procedure codes^a^ or Taiwan health insurance service claims codes^a,b^ used to identify comorbidities at baseline

| Comorbidities | Codes |
| --- | --- |
| Pneumonia | ICD-10-CM diagnosis codes: J12-J17, J18.0-J18.2, A481, A37.91, A22.1, B25.0, B44.0 |
| Influenza | ICD-10-CM diagnosis codes: J09-J11 |
| Acute bronchitis | ICD-10-CM diagnosis codes: J18.9, J20.8, J20.9, J21 |
| COVID-19 | ICD-10-CM diagnosis codes: U07.1, J12.82, Z86.16  Taiwan health insurance service claims codes: XCOVID0001, XCOVID0002, XCOVID0003, E5012C |
| Post COVID condition | ICD-10-CM diagnosis codes: U09.9 |
| Hypertension | ICD-10-CM diagnosis codes: I10-I15, N26.2 |
| Ischemic heart disease or angina | ICD-10-CM diagnosis codes: I20, I24, I25 |
| Myocardial infarction | ICD-10-CM diagnosis codes: I21, I22, I25.2 |
| Coronary revascularization | Coronary artery bypass grafting  ICD-10-CM procedure codes: 0210093, 02100A3, 20100J3, 02100K3, 02100Z3, 0210493, 02104A3, 02104J3, 02104K3, 02104Z3, 021009W, 02100AW, 02100JW, 02100KW, 021049W, 02104AW, 02104JW, 02104KW, 021109W, 02110AW, 02110JW, 02110KW, 021149W, 02114AW, 02114JW, 02114KW, 021209W, 02120AW, 02120JW, 02120KW, 021249W, 02124AW, 02124JW, 02124KW, 021309W, 02130AW, 02130JW, 02130KW, 021349W, 02134AW, 02134JW, 02134KW, 0210098, 0210099, 021009C, 02100A8, 02100A9, 02100AC, 02100J8, 02100J9, 02100JC, 02100K8, 02100K9, 02100KC, 02100Z8, 02100Z9, 02100ZC, 0210498, 0210499, 021049C, 02104A8, 02104A9, 02104AC, 02104J8, 02104J9, 02104JC, 02104K8, 02104K9, 02104KC, 02104Z8, 02104Z9, 02104ZC, 0211098, 0211099, 021109C, 02110A8, 02110A9, 02110AC, 02110J8, 02110J9, 02110JC, 02110J8, 02110K9, 02110KC, 02110Z8, 02110Z9, 02110ZC, 0211498, 0211499, 021149C, 02114A8, 02114A9, 02114AC, 02114J8, 02114J9, 02114JC, 02114K8, 02114K9, 02114KC, 02114Z8, 02114Z9, 02114ZC, 0212098, 0212099, 021209C, 02120A8, 02120A9, 02120AC, 02120J8, 02120J9, 02120JC, 02120K8, 02120K9, 02120KC, 02120Z8, 02120Z9, 02120ZC, 0212498, 0212499, 021249C, 02124A8, 02124A9, 02124AC, 02124J8, 02124J9, 02124JC, 02124K8, 02124K9, 02124KC, 02124Z8, 02124Z9, 02124ZC, 0213098, 0213099, 021309C, 02130A8, 02130A9, 02130AC, 02130J8, 02130J9, 02130JC, 02130K8, 02130K9, 02130KC, 02130Z8, 02130Z9, 02130ZC, 0213498, 0213499, 021349C, 02134A8, 02134A9, 02134AC, 02134J8, 20134J9, 02134JC, 02134K8, 02134K9, 02134KC, 02134Z8, 02134Z9, 02134ZC, 021009F, 02100AF, 02100JF, 02100KF, 021100ZF, 021049F, 02104AF, 02104JF, 02104KF, 02104ZF, 021109F, 02110AF, 02110JAF, 02110KF, 02110ZF, 021149F, 02114AF, 02114JF, 02114KF, 02114ZF, 021209F, 02120AF, 02120JF, 02120KF, 02120ZF, 021249F, 02124AF, 02124JF, 02124KF, 02124ZF, 021309F, 02130AF, 02130JF, 02130KF, 02130ZF, 021349F, 02134AF, 02134JF, 02134KF, 02134ZF, 0211093, 02110A3, 02110J3, 02110K3, 02110Z3, 0211493, 02114A3, 02114J3, 02114K3, 02114Z3, 0212093, 02120A3, 02120J3, 02120K3, 02120Z3, 0212493, 02124A3, 02124J3, 02124K3, 02124Z3, 0213093, 02130A3, 02130J3, 02130K3, 02130Z3, 0213493, 02134A3, 02134J3, 02134K3, 02134Z3, 021K0Z8, 021K0Z9, 021K0ZC, 021K0ZF, 021K0ZW, 021K4Z8, 021K4Z9, 021K4ZC, 021K4ZF, 021K4ZW, 021L09P, 021L09Q, 021L09R, 021L0AP, 021L0AQ, 021L0AR, 021L0JP, 021L0JQ, 021L0JR, 021L0KP, 021L0KQ, 021L0KR, 021L0Z8, 021L0Z9, 021L0ZC, 021L0ZF, 021L0ZP, 021L0ZQ, 021L0ZR, 021L49P, 021L49Q, 021L49R, 021L4AP, 021L4AQ, 021L4AR, 021L4JP, 021L4JQ, 021L4JR, 021L4KP, 021L4KQ, 021L4KR, 021L4Z8, 021L4Z9, 021L4ZC, 021L4ZF, 021L4ZP, 021L4ZQ, 021L4ZR  Taiwan health insurance service claims codes: 68023A, 68023B, 68024A,68024B, 68025A, 68025B, 68053B, 68054B, 68055B, 83064A1, 97901K, 97902A, 97903B, 97906K, 97907A, 97908B, 97911K, 97912A, 97913B, 97916K, 97917A, 97918B, N26002, N26003  Percutaneous transluminal coronary angioplasty  ICD-10-CM procedure codes: 02703ZZ, 02704ZZ, 3E07317, 02713ZZ, 02714ZZ, 02723ZZ, 02724ZZ, 02733ZZ, 02734ZZ, 02C03ZZ, 02C04ZZ, 02C13ZZ, 02C14ZZ, 02C23ZZ, 02C24ZZ, 02C33ZZ, 02C34ZZ  Taiwan health insurance service claims codes: 33076A, 33076B, 33077A, 33077B, 33078A, 33078B, 97511K, 97512A, 97513B, 97516K, 97517A, 97518B, 97521K, 97522A, 97523B |
| Cardiac dysrhythmia | ICD-10-CM diagnosis codes: I46-I49, R00.1 |
| Congestive heart failure | ICD-10-CM diagnosis codes: I50, I09.81, I11.0, I13.0, I13.2 |
| Cerebrovascular disease | ICD-10-CM diagnosis codes: I60-I63, I65-I69, G45, G46 |
| Ischemic stroke | ICD-10-CM diagnosis codes: I63, I65, I66 |
| Hemorrhagic stroke | ICD-10-CM diagnosis codes: I60-I62 |
| Peripheral vascular disease | ICD-10-CM diagnosis codes: I70.2, I70.92, I79.8, I73.9 |
| Diabetes mellitus | ICD-10-CM diagnosis codes: E08-E11, E13 |
| Hyperlipidemia | ICD-10-CM diagnosis codes: E71.30, E75.21, E75.22, E75.24, E75.3, E75.5, E75.6, E77, E78.0-E78.6, E78.70, E78.79, E78.8, E78.9, E88.1, E88.2, E88.9 |
| Thyroid disease | ICD-10-CM diagnosis codes: E01-E03, E05, E06, E89.0 |
| Chronic liver disease | ICD-10-CM diagnosis codes: B16, B17, B18.0-B18.2, B19.1, B19.2, Z22.51, Z22.52, K70, K73, K74.0, K74.3-K74.6, K75.4, K75.81 |
| Gastritis or peptic ulcer disease | ICD-10-CM diagnosis codes: K25-K29, K31.82, K52.81, K92.0-K92.2 |
| Chronic kidney disease | ICD-10-CM diagnosis codes: I12.0, I13.11, I13.2, N18, Z99.2, Z49.31, Z49.32 |
| Rheumatoid arthritis or osteoarthritis | ICD-10-CM diagnosis codes: M05-M08, M12.0, M15-M19, M32, M33, M34.0, M34.1, M34.9, M35.0, M35.8, M35.9, M45, M46.0, M46.1, M46.8, M46.9, M49 |
| Osteoporosis | ICD-10-CM diagnosis codes: M81 |
| Gout | ICD-10-CM diagnosis codes: M10, M1A.0, M1A.2-M1A.9, N20.0 |
| Any cancer other than lung cancer | ICD-10-CM diagnosis codes: C00-C26, C30-C32, C37-C41, C43-C58, C60-C86, C88, C90-C96, C4A, C7A, C7B, D03, D45, Z51.12 |
| Lung cancer | ICD-10-CM diagnosis codes: C33, C34 |
| Dementia | ICD-10-CM diagnosis codes: F01, F02, F03.90, F05, F10.27, G30, G31.01, G31.09, G31.1, G31.83 |
| Seizure | ICD-10-CM diagnosis codes: G40, R56 |
| Depressive disorder | ICD-10-CM diagnosis codes: F32, F33, F34.1, F43.21, F43.23, F06.3, F39 |
| Anxiety disorder | ICD-10-CM diagnosis codes: F40-F42, F44, F45.0-F45.2, F45.8, F45.9, F48.1, F48.8, F48.9, F68.11, F68.8, F99, R45.2, R45.5, R45.6 |
| Psychotic disorder | ICD-10-CM diagnosis codes: F20, F22, F23, F24, F25, F28, F29, F32.3, F33.3, F44.89, F84.0, F84.3, F84.5, F84.8, F84.9, R44.0, R44.2, R44.3 |
| Bipolar disorder | ICD-10-CM diagnosis codes: F30, F31, F32.8, F33.8, F34.8, F34.9, F39 |

COVID, coronavirus disease.

^a^Based on any diagnosis or procedure positions or health insurance services records in the outpatient or inpatient claims.

^b^https://info.nhi.gov.tw/INAE5000/INAE5001S01 Accessed on 10 January, 2025.

**Supplementary Table 4.** Anatomical Therapeutic Chemical (ATC) classification system codes^a^ used to identify medication use at baseline

| Medications | ATC Classification System Codes |
| --- | --- |
| Inhaled short-acting bronchodilators | SABA: R03AC02-R03AC10, R03AC15-R03AC17  SAMA: R03BB01, R03BB02  SABA/SAMA FDC: R03AL01, R03AL02 |
| LABA | R03AC12-R03AC14, R03AC18, R03AC19 |
| LAMA | R03BB04-R03BB08 |
| LABA/ICS FDC | R03AK06-R03AK12, R03AK14 |
| LABA/LAMA FDC | R03AL03-R03AL07, R03AL10 |
| Inhaled corticosteroids | R03BA |
| Systemic bronchodilators | β-2 agonists: R03CC02-R03CC14, R03CC53, R03CC63, R03CC91  Xanthines: R03DA04, R03DA05 |
| Systemic corticosteroids | H02AB, H02BX |
| Oral antibiotics commonly used for COPD exacerbations^b^ | J01CA01, J01CA04, J01CA51, J01CR01, J01CR02, J01CR04, J01DC, J01DD, J01MA, J01FA, J01AA, J01EE01; restricted to an oral form |
| ACEIs or ARBs | C09 |
| Selective β blockers | C07AB, C07BB, C07CB, C07DB, C07EB, C07FB |
| Non-selective β blockers | C07AA, C07AG, C07BA, C07BG, C07CA, C07CG, C07DA, C07EA, C07FX |
| Dihydropyridine CCBs | C08CA |
| Non-dihydropyridine CCBs | C08DA, C08DB |
| Diuretics | C03, C07B, C07C, C07D, C08G |
| Other anti-hypertensive agents | C02 |
| Nitrates | C01DA |
| Anti-arrhythmic agents | C01B |
| Digoxin | C01AA |
| Aspirin | B01AC06, N02BA01 |
| Clopidogrel | B01AC04 |
| Warfarin | B01AA03 |
| Non-vitamin K antagonist oral anticoagulants | B01AE, B01AF |
| Statins | C10AA |
| Fibrates | C10AB |
| Urate-lowering agents | M04A |
| Febuxostat | M04AA03 |
| Insulin | A10A |
| Metformin | A10BA02, A10BD02, A10BD03, A10BD05, A10BD07, A10BD08, A10BD10, A10BD11, A10BD13-A10BD18, A10BD20, A10BD22, A10BD23, A10BD25, A10BD26 |
| Sulfonylureas | A10BB, A10BD01, A10BD02, A10BD04, A10BD06 |
| Glinides | A10BX02, A10BX03, A10BX08, A10BD14 |
| Thiazolidinedione | A10BG, A10BD03-A10BD06, A10BD09, A10BD12, A10BD26 |
| Alpha-glucosidase inhibitors | A10BF, A10BD17 |
| Dipeptidyl peptidase-4 inhibitors | A10BH, A10BD07-A10BD13, A10BD18, A10BD19, A10BD21, A10BD22, A10BD24, A10BD25 |
| Sodium-glucose cotransporter 2 Inhibitors | A10BK, A10BD15, A10BD16, A10BD19-A10BD21, A10BD23-A10BD25 |
| Glucagon-like peptide-1 receptor agonists | A10BJ |
| Thyroid-therapy drugs | H03 |
| Histamine 2 antagonists or Proton pump inhibitors | A02BA, A02BC |
| COX-2 selective NSAIDs | M01AH |
| COX-2 nonselective NSAIDs | M01AA, M01AB, M01AC, M01AE, M01AG, M01AX |
| Anti-epileptics | N03 |
| Anti-depressants: | N06A |
| Anxiolytics | N05B |
| Hypnotics | N05C |
| Anti-psychotics | N05A |

ACEI, angiotensin converting enzyme inhibitor; ARB, angiotensin II receptor blocker; CCB, calcium channel blocker; COPD, chronic obstructive pulmonary disease; COX-2, cyclooxygenase-2; DHP, dihydropyridine; FDC, fixed-dose combinations; ICS, inhaled corticosteroid; LABA, long-acting β_2_ agonists; LAMA, long-acting muscarinic antagonists; NSAID, non-steroidal anti-inflammatory drug; SABA, short-acting β_2_ agonists; SAMA, short-acting muscarinic antagonists.

^a^Based on outpatient or inpatient pharmacy dispensing claims.

^b^Based on the definition in JAMA Intern Med 2023;183:685-695 [10].

**Supplementary Table 5.** International Classification of Diseases, 10^th^ Revision, Clinical Modification (ICD-10-CM) diagnosis or procedure codes^a^ or Taiwan health insurance service claims codes^a,b^ used to measure healthcare services at baseline

| Healthcare services | Codes |
| --- | --- |
| Pneumococcal or influenza vaccination | Pneumococcal vaccination  Taiwan health insurance service claims codes: K000492206  Influenza vaccination  Taiwan health insurance service claims codes: J000113277, K000453265, K000453277, K000523206, K000523265, K000706206, K000889206, X000092206, X000090238, X000090221, X000091221, J000113265, K000901206, J000138206, K001036206, K001126206, X000209206  Case type: D2  Func seq no: 01, 1, IC01 |
| Any outpatient visits | Not applicable |
| Outpatient visits due to COPD | ICD-10-CM diagnosis codes: J41-J44 |
| Outpatient visits due to pneumonia | See ICD-10-CM diagnosis codes for pneumonia in **Supplementary Table 3** |
| Outpatient visits due to cardiovascular disease | See ICD-10-CM diagnosis codes or procedure codes or Taiwan health insurance service claims codes for hypertension, ischemic heart disease or angina, myocardial infarction, coronary revascularization, cardiac dysrhythmia, congestive heart failure, cerebrovascular disease, ischemic stroke, hemorrhagic stroke, peripheral vascular disease, diabetes mellitus, hyperlipidemia in **Supplementary Table 3** |
| Any emergency department visits | Not applicable |
| Emergency department visits due to COPD | ICD-10-CM diagnosis codes: J41-J44 |
| Emergency department visits due to pneumonia | See diagnosis codes for pneumonia in **Supplementary Table 3** |
| Emergency department visits due to cardiovascular disease | See ICD-10-CM diagnosis codes or procedure codes or Taiwan health insurance service claims codes for hypertension, ischemic heart disease or angina, myocardial infarction, coronary revascularization, cardiac dysrhythmia, congestive heart failure, cerebrovascular disease, ischemic stroke, hemorrhagic stroke, peripheral vascular disease, diabetes mellitus, hyperlipidemia in **Supplementary Table 3** |
| Any hospitalizations | Not applicable |
| Hospitalizations due to COPD | ICD-10-CM diagnosis codes: J41-J44 |
| Hospitalizations due to pneumonia | See ICD-10-CM diagnosis codes for pneumonia in **Supplementary Table 3** |
| Hospitalizations due to cardiovascular disease | See ICD-10-CM diagnosis codes or procedure codes or Taiwan health insurance service claims codes for hypertension, ischemic heart disease or angina, myocardial infarction, coronary revascularization, cardiac dysrhythmia, congestive heart failure, cerebrovascular disease, ischemic stroke, hemorrhagic stroke, peripheral vascular disease, diabetes mellitus, hyperlipidemia in **Supplementary Table 3** |
| Spirometry test | Taiwan health insurance service claims codes: 17003B, 17003C, 17006B, 17006C, 17007A, 17007B |
| Echochardiography | Taiwan health insurance service claims codes: 18005B, 18005C, 18006B, 18006C, 18044B |
| Carotid ultrasonography | Taiwan health insurance service claims codes: 20013A, 20013B, 20013C |
| Transcranial ultrasonography | Taiwan health insurance service claims codes: 20026B |
| Lower extremity arterial ultrasonography | Taiwan health insurance service claims codes: 19002BD, 18008B, 18008C |
| 24-hour ECG examination, % | Taiwan health insurance service claims codes: 18019B, 18019C |
| BNP, proBNP, or NT-proBNP test | Taiwan health insurance service claims codes: 12193B, 12193C |

COPD, chronic obstructive pulmonary disease; BNP, B-type natriuretic peptide; ECG, electrocardiography; NT, n-terminal.

^a^Based on any diagnosis or procedure positions or health insurance services records in the outpatient or inpatient claims.

^b^https://info.nhi.gov.tw/INAE5000/INAE5001S01 Accessed on 10 January, 2025.

**Supplementary Table 6.** Baseline characteristics among the eligible cohort

|  | Before matching (n=14,278) | | | After propensity score matching (n=13,424) | | | After high-dimensional propensity score matching (n=12,971) | | |
| --- | --- | --- | --- | --- | --- | --- | --- | --- | --- |
|  | Fluticasone/ umeclidinium/ vilanterol | Beclometasone/ glycopyrronium/ formoterol | Standardized mean difference | Fluticasone/ umeclidinium/ vilanterol | Beclometasone/ glycopyrronium/ formoterol | Standardized mean difference | Fluticasone/ umeclidinium/ vilanterol | Beclometasone/ glycopyrronium/ formoterol | Standardized mean difference |
|  | (n=9,750) | (n=4,528) |  | (n=9,489) | (n=3,935) |  | (n=9,171) | (n=3,800) |  |
|  |  |  |  | (n=3,935^a^) | (n=3,935^a^) |  | (n=3,800^a^) | (n=3,800^a^) |  |
| **Demographics and COPD duration, n (%) or mean (SD)** | | | | | | | | | |
| Age, years, mean (SD) | 70.15 (10.15) | 71.96 (10.98) | -0.171 | 71.06 (10.72) | 70.92 (10.64) | 0.013 | 71.00 (10.65) | 71.00 (10.66) | <0.001 |
| Male, n (%) | 8,733 (89.57) | 3,787 (83.64) | 0.175 | 3,391 (86.18) | 3,383 (85.97) | 0.006 | 3,264 (85.89) | 3,260 (85.79) | 0.003 |
| COPD duration, days, mean (SD)^b^ | 1,649 (934.42) | 1,769 (1045.59) | -0.121 | 1,768 (927.81) | 1,768 (1033.79) | 0.001 | 1752 (947.05) | 1768 (1032.84) | -0.017 |
| **General disease burden, mean (SD)** |  |  |  |  |  |  |  |  |  |
| Charlson Comorbidity Index | 3.10 (2.42) | 3.44 (2.58) | -0.135 | 3.25 (2.50) | 3.21 (2.43) | 0.016 | 3.21 (2.47) | 3.18 (2.41) | 0.010 |
| Claim-based Frailty Index | 0.14 (0.13) | 0.19 (0.17) | -0.306 | 0.16 (0.15) | 0.16 (0.14) | 0.027 | 0.16 (0.14) | 0.16 (0.14) | 0.015 |
| **Comorbidities, n (%)** |  |  |  |  |  |  |  |  |  |
| Pneumonia | 1,329 (13.63) | 902 (19.92) | -0.169 | 642 (16.32) | 624 (15.86) | 0.012 | 561 (14.76) | 580 (15.26) | -0.014 |
| Influenza | 286 (2.93) | 94 (2.08) | 0.055 | 84 (2.13) | 87 (2.21) | -0.005 | 93 (2.45) | 85 (2.24) | 0.014 |
| Acute bronchitis | 3,766 (38.63) | 1,942 (42.89) | -0.087 | 1,535 (39.01) | 1,541 (39.16) | -0.003 | 1,464 (38.53) | 1,450 (38.16) | 0.008 |
| COVID-19 | 284 (2.91) | 341 (7.53) | -0.209 | 192 (4.88) | 197 (5.01) | -0.006 | 186 (4.89) | 194 (5.11) | -0.010 |
| Post COVID condition | 27 (0.28) | 28 (0.62) | -0.051 | 17 (0.43) | 19 (0.48) | -0.008 | 13 (0.34) | 16 (0.42) | -0.013 |
| Hypertension | 5,700 (58.46) | 2,802 (61.88) | -0.070 | 2,350 (59.72) | 2,346 (59.62) | 0.002 | 2,284 (60.11) | 2,243 (59.03) | 0.022 |
| Ischemic heart disease or angina | 2,755 (28.26) | 1,409 (31.12) | -0.063 | 1,200 (30.50) | 1,172 (29.78) | 0.016 | 1,140 (30) | 1,132 (29.79) | 0.005 |
| Myocardial infarction | 314 (3.22) | 187 (4.13) | -0.048 | 150 (3.81) | 143 (3.63) | 0.009 | 133 (3.5) | 129 (3.39) | 0.006 |
| Coronary revascularization | 227 (2.33) | 122 (2.69) | -0.023 | 104 (2.64) | 97 (2.47) | 0.011 | 95 (2.5) | 92 (2.42) | 0.005 |
| Cardiac dysrhythmia | 1,583 (16.24) | 895 (19.77) | -0.092 | 727 (18.48) | 711 (18.07) | 0.011 | 667 (17.55) | 673 (17.71) | -0.004 |
| Congestive heart failure | 1,679 (17.22) | 996 (22.00) | -0.121 | 766 (19.47) | 764 (19.42) | 0.001 | 699 (18.39) | 708 (18.63) | -0.006 |
| Cerebrovascular disease | 1,212 (12.43) | 924 (20.41) | -0.217 | 644 (16.37) | 622 (15.81) | 0.015 | 599 (15.76) | 601 (15.82) | -0.001 |
| Ischemic stroke | 588 (6.03) | 438 (9.67) | -0.136 | 295 (7.50) | 293 (7.45) | 0.002 | 282 (7.42) | 292 (7.68) | -0.010 |
| Hemorrhagic stroke | 123 (1.26) | 149 (3.29) | -0.136 | 83 (2.11) | 66 (1.68) | 0.032 | 74 (1.95) | 76 (2) | -0.004 |
| Peripheral vascular disease | 295 (3.03) | 134 (2.96) | 0.004 | 119 (3.02) | 106 (2.69) | 0.020 | 100 (2.63) | 108 (2.84) | -0.013 |
| Diabetes mellitus | 2,670 (27.38) | 1,398 (30.87) | -0.077 | 1,115 (28.34) | 1,120 (28.46) | -0.003 | 1,093 (28.76) | 1,092 (28.74) | 0.001 |
| Hyperlipidemia | 3,629 (37.22) | 1,651 (36.46) | 0.016 | 1,426 (36.24) | 1,428 (36.29) | -0.001 | 1,380 (36.32) | 1,377 (36.24) | 0.002 |
| Thyroid disease | 307 (3.15) | 192 (4.24) | -0.058 | 145 (3.68) | 135 (3.43) | 0.014 | 144 (3.79) | 135 (3.55) | 0.013 |
| Chronic liver disease | 1,237 (12.69) | 534 (11.79) | 0.027 | 479 (12.17) | 471 (11.97) | 0.006 | 474 (12.47) | 447 (11.76) | 0.022 |
| Gastritis or peptic ulcer disease | 3,560 (36.51) | 1,588 (35.07) | 0.030 | 1,343 (34.13) | 1,350 (34.31) | -0.004 | 1,315 (34.61) | 1,285 (33.82) | 0.017 |
| Chronic kidney disease | 1,578 (16.18) | 823 (18.18) | -0.053 | 698 (17.74) | 652 (16.57) | 0.031 | 642 (16.89) | 628 (16.53) | 0.010 |
| Rheumatoid arthritis or osteoarthritis | 2,149 (22.04) | 1,002 (22.13) | -0.002 | 857 (21.78) | 877 (22.29) | -0.012 | 861 (22.66) | 846 (22.26) | 0.009 |
| Osteoporosis | 393 (4.03) | 184 (4.06) | -0.002 | 159 (4.04) | 157 (3.99) | 0.003 | 147 (3.87) | 146 (3.84) | 0.001 |
| Gout | 1,673 (17.16) | 729 (16.10) | 0.028 | 608 (15.45) | 629 (15.98) | -0.015 | 577 (15.18) | 604 (15.89) | -0.020 |
| Any cancer other than lung cancer | 1,284 (13.17) | 599 (13.23) | -0.002 | 526 (13.37) | 517 (13.14) | 0.007 | 473 (12.45) | 495 (13.03) | -0.017 |
| Lung cancer | 620 (6.36) | 256 (5.65) | 0.030 | 224 (5.69) | 235 (5.97) | -0.012 | 207 (5.45) | 218 (5.74) | -0.013 |
| Dementia | 437 (4.48) | 485 (10.71) | -0.237 | 289 (7.34) | 256 (6.51) | 0.033 | 275 (7.24) | 251 (6.61) | 0.025 |
| Seizure | 49 (0.50) | 53 (1.17) | -0.073 | 27 (0.69) | 23 (0.58) | 0.013 | 26 (0.68) | 22 (0.58) | 0.013 |
| Depressive disorder | 623 (6.39) | 352 (7.77) | -0.054 | 290 (7.37) | 287 (7.29) | 0.003 | 308 (8.11) | 274 (7.21) | 0.034 |
| Anxiety disorder | 1,189 (12.19) | 605 (13.36) | -0.035 | 519 (13.19) | 520 (13.21) | -0.001 | 503 (13.24) | 495 (13.03) | 0.006 |
| Psychotic disorder | 115 (1.18) | 74 (1.63) | -0.039 | 50 (1.27) | 53 (1.35) | -0.007 | 56 (1.47) | 51 (1.34) | 0.011 |
| Bipolar disorder | 145 (1.49) | 81 (1.79) | -0.024 | 58 (1.47) | 67 (1.70) | -0.018 | 69 (1.82) | 65 (1.71) | 0.008 |
| **Medications, n (%)** |  |  |  |  |  |  |  |  |  |
| Inhaled short-acting bronchodilators | 5,985 (61.38) | 2,988 (65.99) | -0.096 | 2,525 (64.17) | 2,505 (63.66) | 0.011 | 2,377 (62.55) | 2,373 (62.45) | 0.002 |
| LABA | 100 (1.03) | 52 (1.15) | -0.012 | 38 (0.97) | 48 (1.22) | -0.024 | 44 (1.16) | 46 (1.21) | -0.005 |
| LAMA | 2,748 (28.18) | 1,374 (30.34) | -0.047 | 1,240 (31.51) | 1,209 (30.72) | 0.017 | 1,167 (30.71) | 1,167 (30.71) | NA |
| LABA/ICS FDC | 3,547 (36.38) | 1,985 (43.84) | -0.153 | 1,709 (43.43) | 1,725 (43.84) | -0.008 | 1,641 (43.18) | 1,648 (43.37) | -0.004 |
| LABA/LAMA FDC | 5,889 (60.40) | 1,895 (41.85) | 0.378 | 1,830 (46.51) | 1,794 (45.59) | 0.018 | 1,744 (45.89) | 1,725 (45.39) | 0.010 |
| ICS | 1,094 (11.22) | 509 (11.24) | -0.001 | 437 (11.11) | 412 (10.47) | 0.020 | 421 (11.08) | 405 (10.66) | 0.014 |
| Systemic bronchodilators | 7,302 (74.89) | 3,471 (76.66) | -0.041 | 3,001 (76.26) | 3,027 (76.93) | -0.016 | 2,942 (77.42) | 2,924 (76.95) | 0.011 |
| Systemic corticosteroids | 6,393 (65.57) | 3,227 (71.27) | -0.123 | 2,713 (68.95) | 2,723 (69.20) | -0.005 | 2,586 (68.05) | 2,603 (68.50) | -0.010 |
| Oral antibiotics commonly used for COPD exacerbations | 6,010 (61.64) | 3,045 (67.25) | -0.117 | 2,536 (64.45) | 2,542 (64.60) | -0.003 | 2,428 (63.89) | 2,417 (63.61) | 0.006 |
| ACEIs or ARBs | 4,346 (44.57) | 2154 (47.57) | -0.060 | 1,813 (46.07) | 1,831 (46.53) | -0.009 | 1,758 (46.26) | 1,759 (46.29) | -0.001 |
| Selective β blockers | 2,431 (24.93) | 1,359 (30.01) | -0.114 | 1,102 (28.01) | 1,094 (27.80) | 0.005 | 1,031 (27.13) | 1,020 (26.84) | 0.007 |
| Non-selective β blockers | 1,107 (11.35) | 546 (12.06) | -0.022 | 458 (11.64) | 438 (11.13) | 0.016 | 429 (11.29) | 419 (11.03) | 0.008 |
| DHP CCBs | 2,822 (28.94) | 1,440 (31.80) | -0.062 | 1,159 (29.45) | 1,160 (29.48) | -0.001 | 1,113 (29.29) | 1,097 (28.87) | 0.009 |
| Non-DHP CCBs | 1,464 (15.02) | 775 (17.12) | -0.057 | 616 (15.65) | 640 (16.26) | -0.017 | 613 (16.13) | 611 (16.08) | 0.001 |
| Diuretics | 2,833 (29.06) | 1,750 (38.65) | -0.204 | 1,358 (34.51) | 1,329 (33.77) | 0.016 | 1,263 (33.24) | 1,238 (32.58) | 0.014 |
| Other anti-hypertensive agents | 1,133 (11.62) | 561 (12.39) | -0.024 | 455 (11.56) | 457 (11.61) | -0.002 | 454 (11.95) | 428 (11.26) | 0.021 |
| Nitrates | 1,788 (18.34) | 894 (19.74) | -0.036 | 726 (18.45) | 747 (18.98) | -0.014 | 713 (18.76) | 708 (18.63) | 0.003 |
| Anti-arrhythmic agents | 951 (9.75) | 594 (13.12) | -0.106 | 446 (11.33) | 436 (11.08) | 0.008 | 407 (10.71) | 403 (10.61) | 0.003 |
| Digoxin | 259 (2.66) | 182 (4.02) | -0.076 | 131 (3.33) | 123 (3.13) | 0.012 | 126 (3.32) | 121 (3.18) | 0.007 |
| Aspirin | 2,669 (27.37) | 1,384 (30.57) | -0.070 | 1,147 (29.15) | 1,139 (28.95) | 0.004 | 1,075 (28.29) | 1,085 (28.55) | -0.006 |
| Clopidogrel | 1,106 (11.34) | 618 (13.65) | -0.070 | 489 (12.43) | 490 (12.45) | -0.001 | 476 (12.53) | 467 (12.29) | 0.007 |
| Warfarin | 115 (1.18) | 79 (1.74) | -0.047 | 53 (1.35) | 58 (1.47) | -0.011 | 51 (1.34) | 59 (1.55) | -0.018 |
| New oral anticoagulants | 677 (6.94) | 380 (8.39) | -0.054 | 301 (7.65) | 297 (7.55) | 0.004 | 293 (7.71) | 291 (7.66) | 0.002 |
| Statins | 3,167 (32.48) | 1,457 (32.18) | 0.007 | 1,274 (32.38) | 1,251 (31.79) | 0.013 | 1,225 (32.24) | 1,226 (32.26) | -0.001 |
| Fibrates | 326 (3.34) | 134 (2.96) | 0.022 | 122 (3.10) | 114 (2.90) | 0.012 | 115 (3.03) | 111 (2.92) | 0.006 |
| Urate-lowering agents | 1,811 (18.57) | 800 (17.67) | 0.024 | 703 (17.87) | 676 (17.18) | 0.018 | 640 (16.84) | 660 (17.37) | -0.014 |
| Febuxostat | 610 (6.26) | 332 (7.33) | -0.043 | 272 (6.91) | 265 (6.73) | 0.007 | 243 (6.39) | 255 (6.71) | -0.013 |
| Insulin | 883 (9.06) | 733 (16.19) | -0.216 | 475 (12.07) | 480 (12.20) | -0.004 | 446 (11.74) | 439 (11.55) | 0.006 |
| Metformin | 1,730 (17.74) | 824 (18.20) | -0.012 | 684 (17.38) | 690 (17.53) | -0.004 | 661 (17.39) | 664 (17.47) | -0.002 |
| Sulfonylureas | 875 (8.97) | 402 (8.88) | 0.003 | 345 (8.77) | 332 (8.44) | 0.012 | 343 (9.03) | 329 (8.66) | 0.013 |
| Glinides | 192 (1.97) | 172 (3.80) | -0.109 | 120 (3.05) | 95 (2.41) | 0.039 | 105 (2.76) | 97 (2.55) | 0.013 |
| Thiazolidinedione | 290 (2.97) | 118 (2.61) | 0.022 | 98 (2.49) | 105 (2.67) | -0.011 | 100 (2.63) | 103 (2.71) | -0.005 |
| Alpha-glucosidase inhibitors | 224 (2.30) | 108 (2.39) | -0.006 | 89 (2.26) | 85 (2.16) | 0.007 | 82 (2.16) | 80 (2.11) | 0.004 |
| Dipeptidyl peptidase-4 inhibitors | 1,180 (12.10) | 670 (14.80) | -0.079 | 512 (13.01) | 503 (12.78) | 0.007 | 500 (13.16) | 499 (13.13) | 0.001 |
| Sodium-glucose cotransporter 2 Inhibitors | 385 (3.95) | 245 (5.41) | -0.069 | 188 (4.78) | 189 (4.80) | -0.001 | 180 (4.74) | 187 (4.92) | -0.009 |
| Glucagon-like peptide-1 receptor agonists | 39 (0.40) | 13 (0.29) | 0.019 | 13 (0.33) | 13 (0.33) | NA | 13 (0.34) | 13 (0.34) | NA |
| Thyroid-therapy drugs | 252 (2.58) | 193 (4.26) | -0.092 | 133 (3.38) | 125 (3.18) | 0.011 | 133 (3.50) | 124 (3.26) | 0.013 |
| Histamine 2 antagonists or proton pump inhibitors | 5,760 (59.08) | 2,784 (61.48) | -0.049 | 2,351 (59.75) | 2,329 (59.19) | 0.011 | 2,238 (58.89) | 2,223 (58.5) | 0.008 |
| COX-2 selective NSAIDs | 1,950 (20.00) | 985 (21.75) | -0.043 | 825 (20.97) | 844 (21.45) | -0.012 | 780 (20.53) | 806 (21.21) | -0.017 |
| COX-2 nonselective NSAIDs | 5,992 (61.46) | 2,485 (54.88) | 0.134 | 2,236 (56.82) | 2,228 (56.62) | 0.004 | 2,126 (55.95) | 2,137 (56.24) | -0.006 |
| Anti-epileptics | 1,571 (16.11) | 893 (19.72) | -0.094 | 688 (17.48) | 685 (17.41) | 0.002 | 686 (18.05) | 650 (17.11) | 0.025 |
| Anti-depressants: | 1,354 (13.89) | 719 (15.88) | -0.056 | 573 (14.56) | 591 (15.02) | -0.013 | 563 (14.82) | 562 (14.79) | 0.001 |
| Anxiolytics | 3,998 (41.01) | 2,082 (45.98) | -0.100 | 1,723 (43.79) | 1,736 (44.12) | -0.007 | 1,672 (44) | 1,640 (43.16) | 0.017 |
| Hypnotics | 2,347 (24.07) | 1,351 (29.84) | -0.130 | 1,078 (27.40) | 1,087 (27.62) | -0.005 | 1,007 (26.5) | 1,007 (26.5) | NA |
| Anti-psychotics | 1,496 (15.34) | 1,092 (24.12) | -0.222 | 765 (19.44) | 765 (19.44) | NA | 713 (18.76) | 709 (18.66) | 0.003 |
| **Healthcare services** |  |  |  |  |  |  |  |  |  |
| ***Vaccination, n (%)*** |  |  |  |  |  |  |  |  |  |
| Pneumococcal or influenza vaccination | 5,763 (59.11) | 3,035 (67.03) | -0.165 | 2,528 (64.24) | 2,524 (64.14) | 0.002 | 2,428 (63.89) | 2,414 (63.53) | 0.008 |
| ***Resource utilization, mean (SD)*** |  |  |  |  |  |  |  |  |  |
| No. of any outpatient visits | 41.81 (26.32) | 40.60 (26.02) | 0.046 | 40.78 (25.78) | 40.52 (26.34) | 0.010 | 40.8 (25.79) | 40.4 (26.33) | 0.015 |
| No. of outpatient visits due to COPD | 11.50 (8.95) | 10.73 (9.56) | 0.083 | 10.94 (8.89) | 10.92 (9.17) | 0.002 | 10.79 (8.69) | 10.92 (9.38) | -0.014 |
| No. of outpatient visits due to pneumonia | 0.46 (2.31) | 0.52 (2.31) | -0.025 | 0.51 (2.49) | 0.49 (2.29) | 0.007 | 0.48 (2.35) | 0.47 (2.16) | 0.005 |
| No. of outpatient visits due to cardiovascular disease^c^ | 12.06 (12.50) | 13.03 (13.34) | -0.075 | 12.43 (12.55) | 12.29 (13.01) | 0.011 | 12.41 (12.46) | 12.21 (12.88) | 0.016 |
| No. of emergency department visits | 1.08 (2.15) | 1.71 (3.07) | -0.238 | 1.35 (2.63) | 1.37 (2.37) | -0.008 | 1.34 (2.67) | 1.3 (2.23) | 0.016 |
| No. of emergency department visits due to COPD | 0.32 (1.04) | 0.51 (1.65) | -0.135 | 0.41 (1.18) | 0.42 (1.13) | -0.006 | 0.41 (1.25) | 0.38 (1.08) | 0.024 |
| No. of emergency department visits due to pneumonia | 0.05 (0.27) | 0.07 (0.34) | -0.069 | 0.05 (0.28) | 0.05 (0.28) | <0.001 | 0.05 (0.28) | 0.05 (0.29) | -0.007 |
| No. of emergency department visits due to cardiovascular disease^c^ | 0.22 (0.79) | 0.36 (0.97) | -0.158 | 0.28 (0.93) | 0.27 (0.83) | 0.003 | 0.27 (0.88) | 0.26 (0.8) | 0.009 |
| No. of any hospitalizations | 0.76 (1.46) | 1.07 (1.77) | -0.191 | 0.89 (1.61) | 0.89 (1.63) | 0.003 | 0.85 (1.57) | 0.83 (1.59) | 0.011 |
| No. of hospitalizations due to COPD | 0.49 (1.11) | 0.68 (1.35) | -0.152 | 0.57 (1.19) | 0.57 (1.21) | 0.004 | 0.54 (1.2) | 0.53 (1.19) | 0.012 |
| No. of hospitalizations due to pneumonia | 0.05 (0.27) | 0.13 (0.48) | -0.201 | 0.08 (0.34) | 0.07 (0.30) | 0.040 | 0.07 (0.3) | 0.07 (0.3) | 0.002 |
| No. of hospitalizations due to cardiovascular disease^c^ | 0.51 (1.18) | 0.79 (1.55) | -0.209 | 0.62 (1.34) | 0.62 (1.39) | -0.005 | 0.61 (1.36) | 0.57 (1.34) | 0.026 |
| ***Spirometry test and cardiovascular systems related examinations, n (%)*** | | | | | | | | | |
| Spirometry test | 7,013 (71.93) | 2,481 (54.79) | 0.361 | 2,401 (61.02) | 2,399 (60.97) | 0.001 | 2,361 (62.13) | 2,331 (61.34) | 0.016 |
| Echochardiography | 3,119 (31.99) | 1,689 (37.30) | -0.112 | 1,381 (35.10) | 1,363 (34.64) | 0.010 | 1,297 (34.13) | 1,286 (33.84) | 0.006 |
| Carotid ultrasonography | 608 (6.24) | 301 (6.65) | -0.017 | 240 (6.10) | 245 (6.23) | -0.005 | 235 (6.18) | 234 (6.16) | 0.001 |
| Transcranial ultrasonography | 367 (3.76) | 182 (4.02) | -0.013 | 140 (3.56) | 144 (3.66) | -0.005 | 135 (3.55) | 139 (3.66) | -0.006 |
| Lower extremity arterial ultrasonography | 85 (0.87) | 48 (1.06) | -0.019 | 35 (0.89) | 30 (0.76) | 0.014 | 36 (0.95) | 34 (0.89) | 0.006 |
| 24-hour ECG examination | 590 (6.05) | 283 (6.25) | -0.008 | 239 (6.07) | 222 (5.64) | 0.018 | 216 (5.68) | 213 (5.61) | 0.003 |
| BNP, proBNP, or NT-proBNP test | 2,558 (26.24) | 1,607 (35.49) | -0.201 | 1,195 (30.37) | 1,226 (31.16) | -0.017 | 1,135 (29.87) | 1,114 (29.32) | 0.012 |

ACEI, angiotensin converting enzyme inhibitor; ARB, angiotensin receptor blocker; BNP, B-type natriuretic peptide; CCB, calcium channel blocker; COPD, chronic obstructive pulmonary disease; COVID, coronavirus disease; COX-2, cyclooxygenase-2; DHP, dihydropyridine; ECG, electrocardiography; FDC, fixed-dose combinations; ICS, inhaled corticosteroids; LABA, long-acting β_2_ agonists; LAMA, long-acting muscarinic antagonists; NA, not applicable; NSAID, non-steroidal anti-inflammatory drug; NT, n-terminal; SD, standard deviation.

^a^One randomly sampled fluticasone/umeclidinium/vilanterol initiator versus one beclometasone/glycopyrronium/formoterol initiator in each matched subset.

^b^COPD duration was defined as the duration from the first recorded date of COPD diagnosis (looking back until 2014/01/01) to the index date.

^c^Cardiovascular disease include hypertension, ischemic heart disease or angina, myocardial infarction, coronary revascularization, cardiac dysrhythmia, congestive heart failure, cerebrovascular disease, ischemic stroke, hemorrhagic stroke, transient ischemic attack, peripheral vascular disease, diabetes mellitus, and hyperlipidemia.

**Supplementary Table 7.** Follow-up, incidence rate, and HR of study outcomes among the eligible cohort based on an intention-to-treat analysis

|  | Before matching | | After propensity score matching | | After high-dimensional propensity score matching | |
| --- | --- | --- | --- | --- | --- | --- |
|  | Fluticasone/ umeclidinium/ vilanterol | Beclometasone/ glycopyrronium/ formoterol | Fluticasone/ umeclidinium/ vilanterol | Beclometasone/ glycopyrronium/ formoterol | Fluticasone/ umeclidinium/ vilanterol | Beclometasone/ glycopyrronium/ formoterol |
|  | ***Severe exacerbations*** | | | | | |
| No. of patients (no. of events) | 9,750 (755) | 4,528 (401) | 9,489 (734) | 3,935 (350) | 9,200 (692) | 3,787 (333) |
| Follow-up days, mean (SD) | 293.26 (114.94) | 242.16 (133.02) | 292.71 (115.34) | 248.97 (131.34) | 292.79 (115.08) | 251.26 (130.87) |
| Incidence rate per 1,000 person-years (95% CI)^a^ | 96.45  (89.81, 103.58) | 133.58  (121.12, 147.31) | 111.81  (100.53, 124.35) | 130.49  (117.51, 144.90) | 107.26  (96.03, 119.80) | 127.83  (114.81, 142.32) |
| HR (95% CI)^b^ | 0.75 (0.66, 0.84) | Reference | 0.86 (0.75, 0.98) | Reference | 0.83 (0.72, 0.95) | Reference |
|  | ***Moderate exacerbations*** | | | | | |
| No. of patients (no. of events) | 9,750 (2,458) | 4,528 (1,172) | 9,489 (2,394) | 3,935 (1,056) | 9,270 (2,304) | 3,827 (1,019) |
| Follow-up days, mean (SD) | 256.11 (132.67) | 210.72 (138.67) | 255.46 (132.96) | 215.71 (138.53) | 256.03 (132.65) | 217.48 (138.34) |
| Incidence rate per 1,000 person-years (95% CI)^a^ | 359.53  (345.6, 374.03) | 448.66  (423.69, 475.1) | 371.55  (349.09, 395.45) | 454.41  (427.81, 482.66) | 365.94  (343.38, 389.97) | 447.18  (420.55, 475.50) |
| HR (95% CI)^b^ | 0.84 (0.78, 0.90) | Reference | 0.84 (0.78, 0.91) | Reference | 0.84 (0.78, 0.90) | Reference |
|  | ***Pneumonia*** | | | | | |
| No. of patients (no. of events) | 9,750 (493) | 4,528 (342) | 9,489 (482) | 3,935 (252) | 9,313 (461) | 3,795 (213) |
| Follow-up days, mean (SD) | 299.83 (110.64) | 248.12 (131.45) | 299.30 (111.08) | 256.58 (128.67) | 299.34 (110.96) | 260.05 (127.67) |
| Incidence rate per 1,000 person-years (95% CI)^a^ | 61.60  (56.39, 67.28) | 111.19  (100.00, 123.61) | 74.76  (65.76, 85.00) | 91.16  (80.58, 103.14) | 68.64  (59.89, 78.66) | 78.83  (68.92, 90.16) |
| HR (95% CI)^b^ | 0.57 (0.49, 0.65) | Reference | 0.83 (0.71, 0.97) | Reference | 0.88 (0.74, 1.04) | Reference |
|  | ***Composite cardiovascular events*** | | | | | |
| No. of patients (no. of events) | 9,750 (177) | 4,528 (98) | 9,489 (172) | 3,935 (78) | 9,349 (164) | 3,851 (67) |
| Follow-up days, mean (SD) | 304.08 (107.79) | 254.81 (128.95) | 303.58 (108.23) | 262.07 (126.47) | 303.49 (108.24) | 264.08 (125.68) |
| Incidence rate per 1,000 person-years (95% CI)^a^ | 21.81  (18.82, 25.27) | 31.02  (25.45, 37.82) | 24.37  (19.50, 30.46) | 27.63  (22.13, 34.49) | 23.70  (18.86, 29.78) | 24.06  (18.94, 30.57) |
| HR (95% CI)^b^ | 0.72 (0.56, 0.92) | Reference | 0.91 (0.69, 1.19) | Reference | 1.00 (0.74, 1.34) | Reference |

CI, confidence interval; HR, hazard ratio; SD, standard deviation.

^a^The incidence rate after propensity score or high-dimensional propensity score matching was weighted by the inverse of the matching ratio.

^b^The HR after propensity score or high-dimensional propensity score matching was stratified on the matching ratio.

**Supplementary Table 8.** Sensitivity analyses for severe and moderate exacerbations comparing fluticasone/umeclidinium/vilanterol with beclometasone/glycopyrrolate/formoterol, after high-dimensional propensity score matching or after propensity score matching with multiple imputation

|  | ***Severe exacerbations*** | | | ***Moderate exacerbations*** | | |
| --- | --- | --- | --- | --- | --- | --- |
|  | Fluticasone/ umeclidinium/ vilanterol | Beclometasone/ glycopyrronium/ formoterol | HR (95% CI)^a^ | Fluticasone/ umeclidinium/ vilanterol | Beclometasone/ glycopyrronium/ formoterol | HR (95% CI)^a^ |
|  | No. of events / No. of patients | |  | No. of events / No. of patients | |  |
| Main analysis | 558/9,171 | 278/3,800 | 0.80 (0.69, 0.93) | 1,945/9,230 | 897/3,822 | 0.80 (0.74, 0.87) |
| Intention-to-treat analysis | 692/9,200 | 333/3,787 | 0.83 (0.72, 0.95) | 2,304/9,270 | 1,019/3827 | 0.84 (0.78, 0.90) |
| Fine-Gray analysis | 558/9,171 | 278/3,800 | 0.81 (0.70, 0.95) | 1,945/9,230 | 897/3,822 | 0.81 (0.75, 0.88) |
| Multiple imputation | 589/9,406 | 308/3,897 | 0.80 (0.69, 0.93) | 2,028/9,447 | 925/3,901 | 0.79 (0.69, 0.91) |

CI, confidence interval; HR, hazard ratio.

^a^The HR after high-dimensional propensity score or propensity score matching was stratified on the matching ratio.

**Supplementary Table 9.** Sensitivity analyses for pneumonia and composite cardiovascular events comparing fluticasone/umeclidinium/vilanterol with beclometasone/glycopyrrolate/formoterol, after high-dimensional propensity score matching or after propensity score matching with multiple imputation

|  | ***Pneumonia*** | | | ***Composite cardiovascular events*** | | |
| --- | --- | --- | --- | --- | --- | --- |
|  | Fluticasone/ umeclidinium/ vilanterol | Beclometasone/ glycopyrronium/ formoterol | HR (95% CI)^a^ | Fluticasone/ umeclidinium/ vilanterol | Beclometasone/ glycopyrronium/ formoterol | HR (95% CI)^a^ |
|  | No. of events / No. of patients | |  | No. of events / No. of patients | |  |
| Main analysis | 363/9,311 | 179/3,815 | 0.85 (0.70, 1.02) | 126/9,355 | 52/3,816 | 0.96 (0.69, 1.35) |
| Intention-to-treat analysis | 461/9,313 | 213/3,795 | 0.88 (0.74, 1.04) | 164/9,349 | 67/3,851 | 1.00 (0.74, 1.34) |
| Fine-Gray analysis | 363/9,311 | 179/3,815 | 0.86 (0.71, 1.04) | 126/9,355 | 52/3,816 | 0.98 (0.70, 1.37) |
| Multiple imputation | 380/9,406 | 208/3,897 | 0.80 (0.66, 0.96) | 134/9,406 | 61/3,897 | 0.90 (0.66, 1.25) |

CI, confidence interval; HR, hazard ratio.

^a^The HR after high-dimensional propensity score or propensity score matching was stratified on the matching ratio.

**Supplementary Table 10.** Number (%) of patients with baseline clinical measures^a^ among the eligible cohort

| No. (%) of patients with information | Fluticasone/ umeclidinium/  vilanterol | Beclometasone/ glycopyrronium/ formoterol | Overall |
| --- | --- | --- | --- |
|  | (n=9,750) | (n=4,528) | (n=14,278) |
|  | Any clinical measures | | |
| ≥ One test result | 8,840 (90.67) | 4,185 (92.42) | 13,025 (91.22) |
|  | Laboratory examinations | | |
| ≥ One test result | 8,320 (85.33) | 4,016 (88.69) | 12,336 (86.40) |
| Eosinophil, % | 6,101 (62.57) | 3,224 (71.20) | 9,325 (65.31) |
| C-reactive protein, mg/dL | 3,568 (36.59) | 2,115 (46.71) | 5,683 (39.80) |
| LDL-cholesterol, mg/dL | 5,004 (51.32) | 2,420 (53.45) | 7,424 (52.00) |
| HbA1c, % | 4,526 (46.42) | 2,310 (51.02) | 6,836 (47.88) |
| GFR or eGFR, mL/min | 7,989 (81.94) | 3,826 (84.50) | 11,815 (82.75) |
|  | Lung function test, respiratory symptoms, blood pressure, or health behavior | | |
| ≥ One test result | 3,207 (32.89) | 1,302 (28.75) | 4,509 (31.58) |
| Predicted post-dose FEV_1_, % | 2,417 (24.79) | 881 (19.46) | 3,298 (23.10) |
| Post-dose FEV_1_/FVC, % | 2,427 (24.89) | 883 (19.50) | 3,310 (23.18) |
| CAT score | 2,878 (29.52) | 1,171 (25.86) | 4,049 (28.36) |
| SBP, mmHg | 2,934 (30.09) | 1,208 (26.68) | 4,142 (29.01) |
| BMI, kg/m^2^ | 2,934 (30.09) | 1,205 (26.61) | 4,139 (28.99) |
| Smoking status | 1,323 (13.57) | 525 (11.59) | 1,848 (12.94) |

BMI, body mass index; CAT, COPD Assessment Test; COPD, chronic obstructive pulmonary disease; eGFR, estimated glomerular filtration rate; FEV_1_, forced expiratory volume in one second; FVC, forced vital capacity; GFR, glomerular filtration rate; HbA1c, glycated hemoglobin; LDL, low-density lipoprotein; SBP, systolic blood pressure.

^a^Collected within 365 days preceding the index date.

**Supplementary Table 11.** Clinical measures among the imputed cohort^a^

|  | Before matching (n=14,278) | | | After propensity score matching (n=13,303) | | |
| --- | --- | --- | --- | --- | --- | --- |
|  | Fluticasone/ umeclidinium/ vilanterol | Beclometasone/ glycopyrronium/ formoterol | Standardized mean difference | Fluticasone/ umeclidinium/ vilanterol | Beclometasone/ glycopyrronium/ formoterol | Standardized mean difference |
|  | (n=9,750) | (n=4,528) |  | (n=9,406) | (n=3,897) |  |
|  |  |  |  | (n=3,935^b^) | (n=3,935^b^) |  |
|  | Laboratory examinations | | | | | |
| Eosinophil, %, mean (SD) | 3.05 (3.19) | 2.96 (3.16) | 0.030 | 2.94 (3.23) | 2.98 (3.10) | -0.012 |
| C-reactive protein, mg/dL, mean (SD) | 1.79 (3.97) | 2.08 (3.99) | -0.073 | 2.00 (4.06) | 1.96 (3.94) | 0.011 |
| LDL-cholesterol, mg/dL, mean (SD) | 100.06 (32.80) | 97.05 (32.85) | 0.092 | 98.88 (33.22) | 98.35 (32.73) | 0.016 |
| HbA1c, %, mean (SD) | 6.14 (1.04) | 6.12 (1.06) | 0.021 | 6.12 (1.04) | 6.11 (1.03) | 0.006 |
| GFR or eGFR, mL/min, mean (SD) | 78.91 (28.27) | 82.04 (32.97) | -0.102 | 80.29 (30.43) | 81.03 (30.92) | -0.024 |
|  | Lung function test, respiratory symptoms, blood pressure, or health behavior | | | | | |
| Predicted post-dose FEV_1_, %, mean (SD) | 59.81 (21.82) | 57.28 (22.04) | 0.115 | 57.44 (22.06) | 57.17 (21.95) | 0.012 |
| Post-dose FEV_1_/FVC, %, mean (SD) | 57.09 (14.22) | 56.75 (14.67) | 0.023 | 56.90 (14.63) | 56.37 (14.59) | 0.036 |
| CAT score, mean (SD) | 11.46 (6.69) | 12.78 (6.64) | -0.198 | 12.27 (6.79) | 12.38 (6.48) | -0.016 |
| SBP, mmHg, mean (SD) | 133.12 (17.72) | 133.67 (18.02) | -0.031 | 133.61 (17.69) | 133.65 (17.83) | -0.003 |
| BMI, kg/m^2^, mean (SD) | 23.85 (4.33) | 23.64 (4.37) | 0.050 | 23.81 (4.43) | 23.76 (4.33) | 0.012 |
| Smoking status, n (%) |  |  |  |  |  |  |
| Current smokers | 2,661 (27.29) | 963 (21.27) | 0.141 | 887 (22.76) | 903 (23.17) | -0.010 |
| Ex-smokers | 5,919 (60.71) | 2,700 (59.63) | 0.022 | 2,359 (60.53) | 2,365 (60.69) | -0.003 |
| Never smokers | 1,170 (12.00) | 865 (19.10) | -0.197 | 651 (16.71) | 629 (16.14) | 0.015 |

BMI, body mass index; CAT, COPD Assessment Test; COPD, chronic obstructive pulmonary disease; eGFR, estimated glomerular filtration rate; FEV_1_, forced expiratory volume in one second; FVC, forced vital capacity; GFR, glomerular filtration rate; HbA1c, glycated hemoglobin; LDL, low-density lipoprotein; SBP, systolic blood pressure; SD, standard deviation.

^a^One randomly sampled dataset from 10 imputed datasets.

^b^One randomly sampled fluticasone/umeclidinium/vilanterol initiator versus one beclometasone/glycopyrronium/formoterol initiator in each matched subset.

**Supplementary Table 12**. Subgroup analyses for severe and moderate exacerbations comparing fluticasone/umeclidinium/vilanterol with beclometasone/glycopyrrolate/formoterol by important patient characteristic, prior dual maintenance medication use, and treatment duration, after high-dimensional propensity score matching

|  | ***Severe exacerbations*** | | | ***Moderate exacerbations*** | | |
| --- | --- | --- | --- | --- | --- | --- |
|  | Fluticasone/ umeclidinium/ vilanterol | Beclometasone/ glycopyrronium/ formoterol | HR (95% CI)^a^ | Fluticasone/ umeclidinium/ vilanterol | Beclometasone/ glycopyrronium/ formoterol | HR (95% CI)^a^ |
|  | No. of events / No. of patients | |  | No. of events / No. of patients | |  |
| Main analysis | 558/9,171 | 278/,3800 | 0.80 (0.69, 0.93) | 1,945/9,230 | 897/3,822 | 0.80 (0.74, 0.87) |
| Charlson comorbidity index | | | | | | |
| ≥2 | 410/6,394 | 197/2,664 | 0.86 (0.72, 1.02) | 1,374/6,407 | 637/2,707 | 0.80 (0.73, 0.88) |
| <2 | 106/2,589 | 59/1,015 | 0.64 (0.46, 0.89) | 482/2,544 | 228/1,026 | 0.80 (0.68, 0.94) |
| Claim-based frailty index | | | | | | |
| ≥0.1 | 316/4,603 | 161/2,009 | 0.82 (0.67, 0.99) | 971/4,551 | 483/2,037 | 0.78 (0.69, 0.87) |
| <0.1 | 223/4,435 | 103/1,709 | 0.82 (0.64, 1.05) | 916/4,473 | 380/1,725 | 0.86 (0.76, 0.97) |
| History of hospitalized COPD exacerbations | | | | | | |
| Yes | 293/2,234 | 147/1,043 | 0.85 (0.69, 1.05) | 603/2,347 | 280/1,062 | 0.81 (0.7, 0.94) |
| No | 232/6,657 | 119/2,604 | 0.70 (0.55, 0.88) | 1,280/6,677 | 586/2,658 | 0.80 (0.72, 0.88) |
| Previous LABA/ICS use | | | | | | |
| Yes | 232/3,226 | 148/1,616 | 0.79 (0.63, 0.98) | 744/3,301 | 411/1,626 | 0.80 (0.71, 0.91) |
| No | 297/5,629 | 129/2,106 | 0.79 (0.64, 0.99) | 1,127/5,721 | 449/2,128 | 0.82 (0.73, 0.92) |
| Previous LABA/LAMA use | | | | | | |
| Yes | 351/5,244 | 146/1,664 | 0.80 (0.65, 0.97) | 1,173/5,311 | 397/1,701 | 0.85 (0.75, 0.95) |
| No | 186/3,746 | 118/2,029 | 0.86 (0.68, 1.10) | 713/3,724 | 475/2,043 | 0.75 (0.66, 0.84) |
| Treatment duration | | | | | | |
| 1-90 days | 257/9,248 | 166/3,766 | 0.68 (0.56, 0.84) | 1,102/9.215 | 563/3,845 | 0.78 (0.70, 0.87) |
| 91-365 days | 280/6,824 | 111/2,648 | 0.98 (0.78, 1.23) | 812/6,184 | 324/2,318 | 0.86 (0.74, 0.98) |

CI, confidence interval; COPD, chronic obstructive pulmonary disease; HR, hazard ratio; ICS, inhaled corticosteroids; LABA, long-acting β_2_ agonists; LAMA, long-acting muscarinic antagonists.

^a^The HR after high-dimensional propensity score o matching was stratified on the matching ratio.

**Supplementary Table 13**. Subgroup analyses for pneumonia and composite cardiovascular events comparing fluticasone/umeclidinium/vilanterol with beclometasone/glycopyrrolate/formoterol by important patient characteristic, prior dual maintenance medication use, and treatment duration, after high-dimensional propensity score matching

|  | ***Pneumonia*** | | | ***Composite cardiovascular events*** | | |
| --- | --- | --- | --- | --- | --- | --- |
|  | Fluticasone/ umeclidinium/ vilanterol | Beclometasone/ glycopyrronium/ formoterol | HR (95% CI)^a^ | Fluticasone/ umeclidinium/ vilanterol | Beclometasone/ glycopyrronium/ formoterol | HR (95% CI)^a^ |
|  | No. of events / No. of patients | |  | No. of events / No. of patients | |  |
| Main analysis | 363/9,311 | 179/3,815 | 0.85 (0.70, 1.02) | 126/9,355 | 52/3,816 | 0.96 (0.69, 1.35) |
| Charlson comorbidity index | | | | | | |
| ≥2 | 283/6,479 | 131/2,671 | 0.89 (0.72, 1.10) | 99/6,449 | 42/2,683 | 0.95 (0.65, 1.39) |
| <2 | 48/2,544 | 29/1,020 | 0.63 (0.39, 1.01) | 18/2,442 | 7/1,032 | 1.02 (0.41, 2.56) |
| Claim-based frailty index | | | | | | |
| ≥0.1 | 230/4,615 | 117/2,001 | 0.84 (0.66, 1.06) | 80/4,645 | 32/1,982 | 1.01 (0.66, 1.54) |
| <0.1 | 100/4,395 | 49/1,697 | 0.76 (0.53, 1.09) | 37/4,458 | 14/1,722 | 0.93 (0.49, 1.76) |
| History of hospitalized COPD exacerbations | | | | | | |
| Yes | 164/2,331 | 80/1,065 | 0.91 (0.69, 1.21) | 33/2,296 | 22/1,031 | 0.64 (0.37, 1.13) |
| No | 171/6,711 | 79/2,667 | 0.77 (0.58, 1.01) | 77/6,580 | 26/2,634 | 1.04 (0.66, 1.65) |
| Previous LABA/ICS use | | | | | | |
| Yes | 137/3,316 | 67/1,611 | 0.99 (0.73, 1.34) | 40/3,280 | 21/1,597 | 0.86 (0.50, 1.48) |
| No | 201/5,733 | 86/2,096 | 0.82 (0.63, 1.06) | 75/5,634 | 24/2,103 | 1.09 (0.68, 1.76) |
| Previous LABA/LAMA use | | | | | | |
| Yes | 230/5,360 | 83/1,689 | 0.89 (0.69, 1.16) | 67/5,255 | 17/1,715 | 1.25 (0.72, 2.17) |
| No | 115/3,717 | 84/2,011 | 0.73 (0.55, 0.98) | 45/3,726 | 28/2,063 | 0.81 (0.50, 1.33) |
| Treatment duration | | | | | | |
| 1-90 days | 140/9,255 | 79/3,792 | 0.80 (0.60, 1.06) | 60/9,328 | 28/3,718 | 0.89 (0.56, 1.42) |
| 91-365 days | 208/6,979 | 89/2,715 | 0.93 (0.72, 1.20) | 62/7,110 | 22/2,740 | 1.07 (0.64, 1.77) |

CI, confidence interval; COPD, chronic obstructive pulmonary disease; HR, hazard ratio; ICS, inhaled corticosteroids; LABA, long-acting β_2_ agonists; LAMA, long-acting muscarinic antagonists.

^a^The HR after high-dimensional propensity score matching was stratified on the matching ratio.

**Supplementary Figure 1.** Cohort identification

| Patients with a COPD diagnosis during 2019/01/01-2022/12/31 (n=1,337,947) | |  | |
| --- | --- | --- | --- |
|  |  |  |  |
|  |  | Excluded due to   - Age <40 years or >100 years on the COPD diagnosis date (n=181,045) - Ambiguous information on age and sex (n=12) - Patients who did not receive single-inhaler triple therapies after the COPD diagnosis date during 2019/07/01-2022/12/31 (n=1,127,459) | |
|  |  |  |  |
|  |  |  |  |
| Patients with COPD who received single-inhaler triple therapies during 2019/07/01~2022/12/31 (n=29,431) | |  |  |
|  |  |  |  |
|  |  | Excluded due to   - Patients who did not have interactions with the healthcare systems   within 365 days preceding the index date^a^ (n=32)   - Patients who received single-inhaler triple therapies within 365 days preceding the index date^a^ (n=0) - Patients who simultaneously received two types of single-inhaler triple therapies on the index date^a^ (n=1) - Patients who simultaneously received inhaled medications that contain any components of the opposite single-inhaler triple therapies on the index date^a^ (n=176) - Patients with an asthma diagnosis within 365 days preceding or on the index date^a^ (n=14,910) - Patients who had death records preceding or on the index date^a^ (n=31) - Patients who were censored on the index date^a^ (n=34) | |
|  |  |  |  |
|  |  |  |  |
| Eligible initiation cohort (n=14,278)   - Fluticasone/umeclidinium/vilanterol (n=9,750) - Beclometasone/glycopyrrolate/formoterol (n=4,528) | |  |  |

COPD, chronic obstructive pulmonary disease.

^a^The date of the first dispensing of a single-inhaler triple therapy after a diagnosis of COPD.

**Supplementary Figure 2.** Propensity score and high-dimensional propensity score plots among the eligible cohort

1. Before matching

| 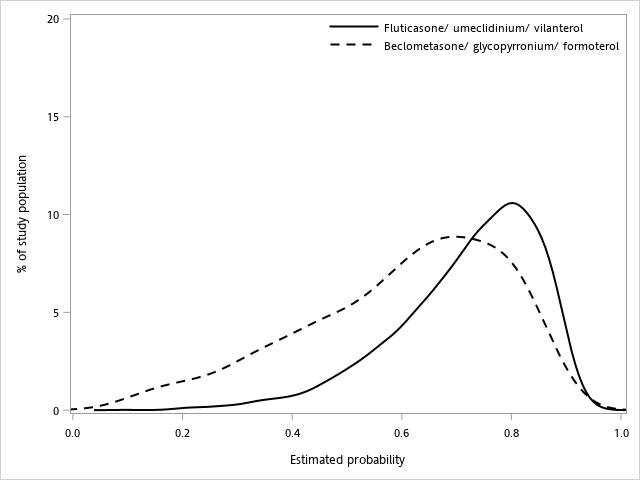 |
| --- |

1. After propensity score matching^a^

| 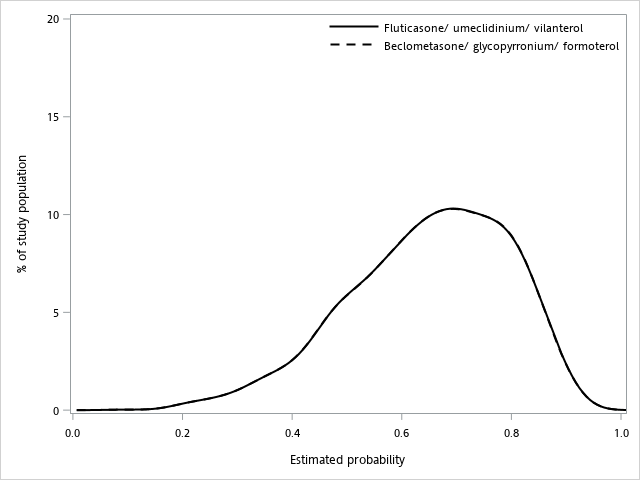 |
| --- |

1. After high-dimensional propensity score matching^a^

| 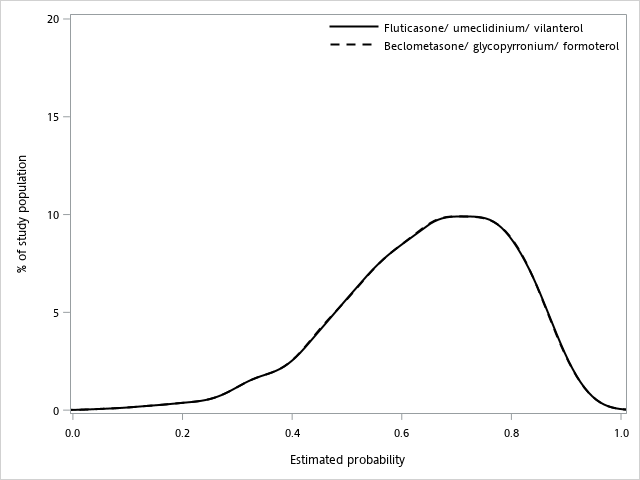 |
| --- |

^a^One randomly sampled fluticasone/umeclidinium/vilanterol initiator versus one beclometasone/glycopyrronium/formoterol initiator in each matched subset.

**Supplementary Figure 3.** Cumulative incidence plots of study outcomes among the eligible cohort, after high-dimensional propensity score matching^a^

(a) Severe exacerbations

| 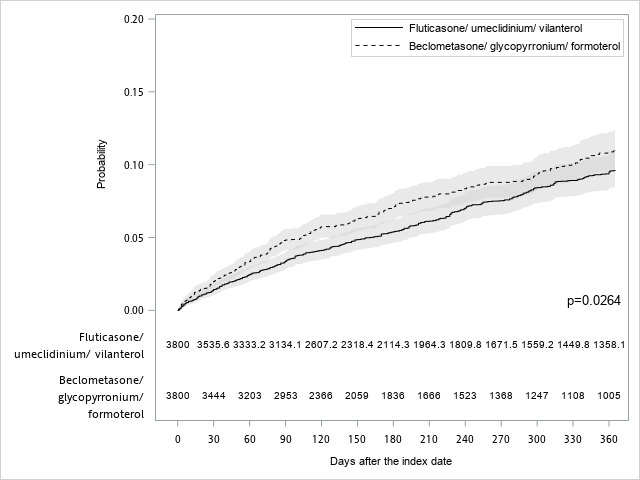 |
| --- |

(b) Moderate exacerbations

| 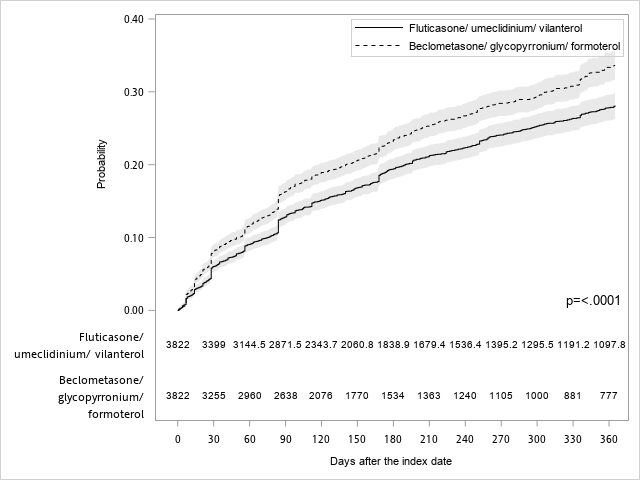 |
| --- |

(c) Pneumonia

| 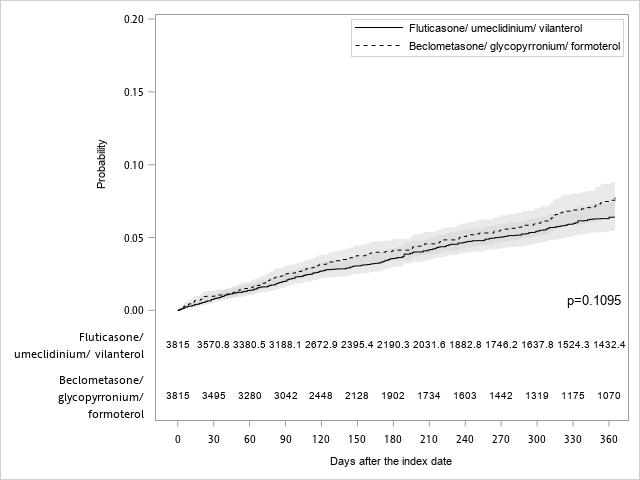 |
| --- |

(c) Composite cardiovascular events

| 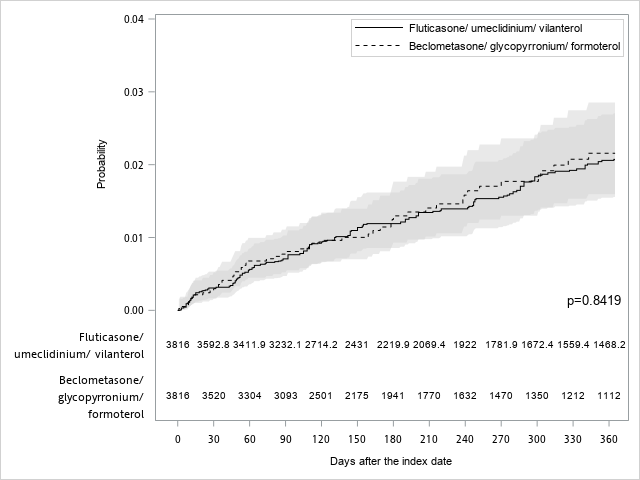 |
| --- |

^a^The cumulative incidence plots after high-dimensional propensity score matching were weighted by the inverse of the matching ratio.

**Supplementary References**

1. Stein BD, Bautista A, Schumock GT, et al. The validity of International Classification of Diseases, Ninth Revision, Clinical Modification diagnosis codes for identifying patients hospitalized for COPD exacerbations. Chest. 2012;141(1):87-93. <https://doi.org/10.1378/chest.11-0024>
2. Rothnie KJ, Müllerová H, Hurst JR, Smeeth L, Davis K, Thomas SL, et al. Validation of the recording of acute exacerbations of COPD in UK primary care electronic healthcare records. PLoS One. 2016;11(3):e0151357. <https://doi.org/10.1371/journal.pone.0151357>
3. Drahos J, Vanwormer JJ, Greenlee RT, et al. Accuracy of ICD-9-CM codes in identifying infections of pneumonia and herpes simplex virus in administrative data. Ann Epidemiol. 2013;23(5):291-293. <https://doi.org/10.1016/j.annepidem.2013.02.005>
4. Coloma PM, Valkhoff VE, Mazzaglia G, et al. Identification of acute myocardial infarction from electronic healthcare records using different disease coding systems: a validation study in three European countries. BMJ Open. 2013;3(6):e002862. <https://doi.org/10.1136/bmjopen-2013-002862>
5. Sundbøll J, Adelborg K, Munch T, et al. Positive predictive value of cardiovascular diagnoses in the Danish National Patient Registry: a validation study. BMJ Open. 2016;6(11):e012832. <https://doi.org/10.1136/bmjopen-2016-012832>
6. Bosco-Lévy P, Duret S, Picard F, et al. Diagnostic accuracy of the International Classification of Diseases, Tenth Revision, codes of heart failure in an administrative database. Pharmacoepidemiol Drug Saf. 2019;28(2):194-200. <https://doi.org/10.1002/pds.4690>
7. Hennessy S, Leonard CE, Freeman CP, et al. Validation of diagnostic codes for outpatient-originating sudden cardiac death and ventricular arrhythmia in Medicaid and Medicare claims data. Pharmacoepidemiol Drug Saf. 2010;19(6):555-562. <https://doi.org/10.1002/pds.1869>
8. Hennessy S, Leonard CE, Newcomb C, et al. Cisapride and ventricular arrhythmia. Br J Clin Pharmacol. 2008;66(3):375-385. <https://doi.org/10.1111/j.1365-2125.2008.03249.x>
9. Hsieh MT, Hsieh CY, Tsai TT, et al. Performance of ICD-10-CM diagnosis codes for identifying acute ischemic stroke in a National Health Insurance claims database. Clin Epidemiol. 2020;12:1007-1013. <https://doi.org/10.2147/CLEP.S273853>
10. Feldman WB, Avorn J, Kesselheim AS, et al. Chronic obstructive pulmonary disease exacerbations and pneumonia hospitalizations among new users of combination maintenance inhalers. JAMA Intern Med 2023;183:685-695. <https://doi.org/10.1001/jamainternmed.2023.1245>
